# Necro-mosaic: a lesion-aware framework for testing programmed cell-death-associated mechanisms in tuberculosis

**DOI:** 10.3389/fimmu.2026.1933352

**Published:** 2026-10-05

**Authors:** Qing Zhang, De Chang

**Affiliations:** 1 Department of Pulmonary and Critical Care Medicine at the Seventh Medical Center Chinese People’s Liberation Army (PLA) General Hospital, Beijing, China; 2 College of Pulmonary Critical Care Medicine of the Eighth Medical Center Chinese People's Liberation Army (PLA) General Hospital, Beijing, China

**Keywords:** ferroptosis, granuloma, host-directed therapy, lesion heterogeneity, neutrophil extracellular traps, PANoptosis, programmed cell death, tuberculosis

## Abstract

Tuberculosis (TB) can progress despite substantial systemic immune activation, indicating that protection and immunopathology are shaped not only by the magnitude of host responses but also by their organization within individual lesions. TB granulomas are heterogeneous tissue ecosystems in which cellular composition, necrosis, vascular access, extracellular-matrix remodeling, metabolic stress, and local drug exposure vary across lesions and over time. Spatial and single-cell technologies now enable these features to be investigated at increasing resolution; however, spatial or molecular association alone does not establish mechanism, causality, or therapeutic relevance. Here, we propose the Necro-Mosaic framework as a hypothesis-generating, lesion-aware approach for investigating programmed cell death (PCD)-associated processes in TB. The framework is neither a mutually exclusive taxonomy nor a clinical classification system. It considers three non-exclusive, mechanistically framed axes: F-axis, involving ferroptosis-associated lipid-peroxidation injury and membrane damage; P-axis, involving coordinated inflammatory cell-death activity with PANoptosis-like features; and N-axis, involving neutrophil-associated extracellular activity, including NET-associated structures and effector processes. The anatomical distribution, co-occurrence, temporal sequence, and biological consequences of these axis-associated processes remain empirical questions. A single lesion may therefore exhibit one axis-associated pattern, several overlapping patterns, or no interpretable pattern. We distinguish pathway capacity, pathway engagement, and mechanism-specific execution and specify the measurements and controls required to support each level of inference *in situ*. The framework also defines evidence as established, emerging, or hypothesis-generating according to the strength, reproducibility, resolution, and causal support of the available data. We propose falsifiable predictions to determine whether F-, P-, or N-axis-associated processes act as drivers, amplifiers, or downstream correlates of destructive lesion evolution. Although lesion-resolved host-directed therapy (HDT) is an important long-term objective, current evidence does not support assigning lesion-level mechanistic profiles from blood alone or using these axes to select clinical treatment. Progress will require prospective longitudinal studies, lesion-anchored multimodal validation, perturbation and rescue experiments, spatial pharmacology, and explicit assessment of antimicrobial-preservation and safety risks. By prioritizing testable mechanisms over fixed spatial categories, Necro-Mosaic provides a structured framework for determining how PCD-associated processes may contribute to TB lesion heterogeneity and for identifying which observations are sufficiently validated to support future translational studies.

## 1 Introduction

Tuberculosis (TB) remains a major cause of morbidity and mortality worldwide despite the availability of effective antimicrobial therapy (1). A central challenge in TB immunology is that robust systemic immune activation does not reliably predict sterilizing protection. Patients can exhibit substantial antigen-specific T-cell responses and inflammatory activity while continuing to develop persistent infection, necrosis, cavitation, transmission, or relapse after treatment (1, 2). This apparent paradox indicates that the effectiveness of immunity cannot be inferred from systemic inflammatory measurements alone, because local immune organization and lesion-specific host–pathogen interactions may determine whether immune activation is translated into bacterial containment or tissue injury (2–4). Within this local logic, upstream coordination nodes—such as purinergic ATP signaling (e.g., ATP–P2X7) shaped by ectonucleotidase activity (e.g., CD39)—can influence the balance between containment versus bacterial release/transfer, and thereby modulate whether inflammatory cell-death programs translate into lesion progression (5).

A growing body of work shows that TB is organized at the level of individual lesions. Granulomas within the same host can differ markedly in bacterial burden, cellular composition, necrotic morphology, vascularization, fibrosis, metabolic state, and drug penetration (2, 3, 6). Consequently, a patient may simultaneously harbor lesions with different biological trajectories. The terms restrictive and permissive are used here as dynamic descriptors of lesion behavior rather than fixed histopathological categories. Restrictive lesions are those in which bacterial growth and destructive remodeling remain limited over time; permissive lesions are those in which bacterial persistence, necrotic expansion, tissue destruction, or cavitation progresses. These states represent poles along a continuum rather than mutually exclusive patient categories, consistent with longitudinal observations in experimental TB and macaque models (6, 7). They should be operationalized using longitudinal microbiological, imaging, and pathological measures whenever possible. Cross-sectional observations should be interpreted as stage-associated unless temporal validation is available (1, 6).

Spatial organization is likely to influence this continuum. Lesion architecture can affect which cells encounter one another, how soluble mediators diffuse, where oxygen and nutrients are available, how extracellular matrix (ECM) remodeling proceeds, and whether antimicrobials achieve effective local exposure (3, 8, 9). However, spatial proximity should not be equated with functional interaction, and a spatially enriched molecular signature should not be interpreted as causal without temporal or perturbational evidence. Spatial transcriptomic co-occurrence, in particular, may reflect mixed cellular neighborhoods rather than same-cell pathway activation (10). Importantly, spatial association should therefore be treated as an empirical observation to be tested rather than as a defining property of mechanistic modules (10, 11).

Recent advances in multiplex imaging, spatial transcriptomics, spatial proteomics, and single-cell profiling have made it possible to map cellular states and neighborhoods within human and experimental TB lesions (3, 10–12). These approaches provide an opportunity to move beyond the question of which immune cells are present toward the questions of where they are located, what they are doing locally, and whether their local activity precedes or follows lesion deterioration. Their limitations must also be recognized. Human resection samples are commonly obtained from patients with advanced, drug-resistant, or treatment-refractory disease and may therefore overrepresent destructive lesions (1, 6). In addition, lesion architecture, hypoxia, necrosis, and neutrophil biology differ substantially among humans, nonhuman primates, rabbits, guinea pigs, zebrafish, conventional mouse strains, and C3HeB/FeJ mice (6, 13). Evidence from one system should not be assumed to apply directly to another; cross-system interpretation requires explicit attention to host species, lesion stage, tissue source, treatment status, and assay resolution (1, 6).

Existing TB endotype frameworks have primarily stratified patients using blood transcriptional profiles, immune-cell abundance, inflammatory mediators, metabolic features, or clinical characteristics (14–16). These approaches provide important systemic information but do not fully resolve lesion-level heterogeneity. Blood-based signatures may have diagnostic, prognostic, or treatment-monitoring value, but their ability to identify mechanisms operating within individual lesions requires prospective calibration against lesion-resolving imaging and tissue-based measurements (14–16). The purpose of the present Review is not to replace those frameworks. Instead, we introduce Necro-Mosaic as a lesion-aware vocabulary for evaluating whether selected cell-death-associated processes contribute to local tissue damage, bacterial persistence, or lesion remodeling.

Necro-Mosaic focuses on three candidate mechanistic axes: ferroptosis-associated lipid peroxidation and membrane damage; PANoptosis-like inflammatory cell-death convergence; and NET-associated neutrophil extracellular effector activity. Ferroptosis is a regulated cell-death pathway involving iron-dependent lipid peroxidation, although lipid oxidation alone is not sufficient to establish mechanism-specific ferroptotic execution (17, 18). PANoptosis has been proposed as coordinated crosstalk among pyroptotic, apoptotic, and necroptotic pathways, but its molecular organization and the generalizability of a discrete PANoptosome remain debated across biological contexts (19, 20). NET-associated extracellular activity involves neutrophil-derived extracellular chromatin and granule proteins; however, neutrophil abundance and lineage-associated markers do not establish NET formation or NETotic cell death (21). Importantly, perturbational evidence in this context also supports intervention plausibility—i.e., targeting relevant neutrophil effector pathways may shift NET-related outputs—while lesion-resolved temporal validation is still required.

These axes are not equivalent biological entities, are not mutually exclusive, and should not be treated as a closed taxonomy. Crucially, the axes are not defined by—or assumed to be confined to—fixed anatomical compartments or predetermined spatial niches. Any spatial distribution, overlap, and temporal ordering must be established empirically through lesion-resolved measurements (3, 10, 11). In practice, a single lesion may show one axis, several overlapping axes, or an indeterminate or non-interpretable axis-associated pattern when execution-level evidence is insufficient or when pathway engagement cannot be distinguished from capacity alone. The possibility of mixed and indeterminate lesion profiles is therefore incorporated explicitly into the framework rather than treated as an anomaly.

The Review further emphasizes evidence tiering. For each axis, observations consistent with pathway capacity, such as expression of susceptibility markers or the presence of relevant cell populations, must be separated from evidence of pathway engagement, such as biochemical or protein-level activation signatures, and from mechanism-specific execution, such as *in situ* execution-level features supported by appropriate structural, temporal, functional, exclusion, and perturbational controls (19, 20, 22). Spatial co-localization and transcriptomic neighborhood enrichment alone indicate where pathway-relevant biology is present, but they do not, by themselves, establish engagement or execution (10, 11). Similarly, lipid-peroxidation readouts can support F-axis-associated injury or engagement but do not independently prove ferroptosis (17). Same-cell or protein-level evidence of multiple activated branches is required before coordinated P-axis activity can be distinguished from mixed-cell neighborhood co-occurrence (19, 20). NET-associated extracellular structures require extracellular DNA structurally co-localized with citrullinated histone H3 and relevant neutrophil proteins, together with appropriate compartmental and negative controls; these structures should not automatically be interpreted as NETotic cell death (21). Axis-specific minimum validation requirements are summarized in **Supplementary Tables S1**-**S3**.

The central proposition of this Review is deliberately limited and falsifiable: cell-death-associated processes may contribute to lesion heterogeneity, but their spatial distribution, temporal sequence, overlap, and causal consequences must be measured rather than assumed. We use the Necro-Mosaic framework to distinguish what is established, what is emerging, and what remains hypothetical; to define minimum standards for pathway inference; and to specify experiments that could disconfirm the proposed mechanisms. This distinction is particularly important because cell-death-associated signals may represent causal drivers, amplifiers of established injury, compensatory responses, or downstream correlates. Such alternatives cannot be resolved by marker abundance or spatial co-localization alone and require temporal linkage, mechanism-specific execution measurements, and selective perturbation (11, 23).

Any eventual translation to host-directed therapy (HDT) will require lesion-resolved pharmacology, demonstration of benefit beyond conventional antimicrobial therapy, and careful assessment of risks to protective immunity. Existing precision-medicine and stratified-HDT proposals emphasize the potential value of biomarker-guided treatment, but they do not establish that blood-based measurements can identify mechanisms within individual lesions or that lesion-level axis assignment improves treatment outcomes (16, 24). Spatial pharmacology is also essential because drug distribution within TB lesions can be heterogeneous, and drug presence does not necessarily establish effective target engagement (8, 9). These requirements remain beyond what systemic biomarkers or spatial association alone can currently justify. In addition, any intervention that modifies inflammatory, oxidative, iron-handling, neutrophil, extracellular-chromatin, or cell-death-associated pathways must be assessed for possible impairment of protective antimycobacterial immunity. The risk associated with excessive immunomodulation is illustrated by TNF blockade, which can increase the risk of TB reactivation and dissemination (25).

### 1.1 Evidence-status rubric

To avoid equating biological plausibility with clinical validation, the evidence-status labels used throughout this Review are defined as follows. “Established” refers to a reproducible observation, measurement principle, or clinical phenomenon supported by multiple independent studies, with at least one appropriate primary evidence system and a clearly bounded inference. Established does not necessarily imply lesion-level causality, mechanism-specific execution, or therapeutic actionability. “Emerging” refers to convergent but incomplete evidence, commonly involving limited human lesion data, cross-sectional association, early longitudinal evidence, or supportive animal/ex vivo studies without complete replication, mechanism-specific execution evidence, or causal perturbation. “Hypothesis-generating” refers to a proposed lesion-level mechanism, spatial relationship, causal role, biomarker application, or treatment-selection implication for which direct human validation and/or temporal, perturbational, rescue, or clinical-outcome evidence is absent.

These labels are assigned according to the evidence system, including human lesion, human blood or fluid, animal, ex vivo, or *in vitro* evidence; assay specificity; spatial and cellular resolution; temporal linkage; replication; and perturbational support. They do not replace the separate capacity–engagement–mechanism-specific-validation framework. A finding may therefore be established at the level of cellular identity, measurement principle, or clinical imaging while remaining emerging or hypothesis-generating at the level of lesion-specific mechanism, causal contribution, or HDT allocation. The evidence-status rubric is an operational framework developed for this Review and should be applied to the specific inference being made rather than to an entire biological process without qualification.

### 1.2 Literature identification and scope

This article is a narrative review informed by an iterative, non-systematic literature search rather than a systematic review or meta-analysis. PubMed, Web of Science, and Scopus were searched from database inception to August 11, 2026. Searches combined terms for tuberculosis and granulomas (“tuberculosis,” “Mycobacterium tuberculosis,” “granuloma”) with terms for spatial biology (“spatial transcriptomics,” “spatial proteomics,” “single-cell,” “multiplex imaging”), cell-death-associated processes (“ferroptosis,” “PANoptosis,” “pyroptosis,” “necroptosis,” “neutrophil extracellular traps,” “NETosis”), lesion context (“hypoxia,” “cavitation,” “matrix metalloproteinase,” “drug penetration,” “MALDI mass spectrometry imaging”), and host-directed therapy (“host-directed therapy,” “metformin,” “statin,” “vitamin D,” “everolimus,” “CC-11050,” and “corticosteroid”).

English-language peer-reviewed primary studies were prioritized when they provided human TB lesion/resection, biopsy, sputum, bronchoalveolar-lavage, or clinical-trial evidence; clinically relevant animal-model evidence; or mechanistic M. tuberculosis infection data directly relevant to a stated inference. Reviews were used for contextual background and citation tracking, but were not used as the sole support for substantive mechanistic or clinical claims when primary evidence was available. Studies were excluded when they lacked a TB-relevant system, did not contain interpretable primary data for the claim being made, or were superseded by a directly relevant primary report. Preprints were identified where relevant but were not used as the sole support for substantive conclusions and are labeled as preprints at the point of citation.

Records were screened iteratively by title, abstract, and full text according to relevance to the conceptual question, evidence system, assay resolution, and ability to distinguish pathway capacity, pathway engagement, mechanism-specific execution, temporal linkage, or perturbational support. Because this was a non-systematic review, no formal PRISMA workflow or quantitative evidence synthesis was undertaken. When findings differed across species, lesion stages, treatment status, or measurement platforms, this heterogeneity was reported rather than interpreted as confirmatory replication. The purpose of this Review is to define a falsifiable lesion-aware framework, critically assess the current evidence, and identify experiments required to evaluate the proposed mechanisms.

### 1.3 What necro-mosaic adds beyond an evidence rubric

The capacity–engagement–execution hierarchy is a general evidentiary discipline and is not, by itself, unique to TB. Necro-Mosaic adds a lesion-level hypothesis structure: it asks whether non-exclusive, spatially and temporally heterogeneous combinations of axis-associated processes, local tissue constraints, bacterial localization, clearance/resolution conditions, and local drug exposure are associated with divergent lesion trajectories. Thus, “mosaic” does not denote fixed anatomical tiles or deterministic compartment-to-module assignments. It denotes the possibility that different regions and interfaces within and across TB lesions contain partially overlapping, evolving, or indeterminate mechanistic patterns whose relationship to tissue injury, bacterial control, and treatment response must be tested empirically. Ultimately, while a standard evidence rubric dictates how to rigorously evaluate cell death in any tissue, Necro-Mosaic proposes a specific, testable biological hypothesis: that the spatial distribution, temporal overlap, and local microenvironmental constraints of distinct F-, P-, and N-axis processes are hypothesized to actively drive TB lesions toward restrictive or permissive trajectories.

## 2 TB granuloma as a spatial immune ecosystem and lesion spectrum

### 2.1 Granulomas are dynamic immune organs, not inert barriers

Accumulating evidence from animal models and human resection or biopsy studies indicates that TB granulomas are dynamic immune microenvironments rather than inert physical barriers (1). Within granulomas, host cells are continuously recruited, differentiate into specialized functional states, and undergo regulated cell-death processes that can reshape cellular composition, inflammatory signaling, tissue architecture, and bacterial localization (26). However, causal inference in humans remains limited because tissue samples are frequently obtained from advanced, drug-resistant, or treatment-refractory disease. Direct lesion-resolved and longitudinal mapping of mechanism-specific cell-death execution in human TB therefore remains uncommon (1, 26).

In parallel, studies using spatial profiling, animal models, and *in vitro* systems indicate that stromal and extracellular-matrix (ECM) remodeling can progressively reconfigure lesion architecture, while metabolic adaptation modifies cellular functions in response to local nutrient availability, oxygen tension, lipid exposure, and other stressors (27, 28, preprint). These observations support the biological plausibility of lesion-specific metabolic regulation, but they do not by themselves establish that a particular metabolic state causes lesion progression. In humans, execution-level metabolic causality is still inferred primarily from cross-sectional profiling or indirect functional measurements rather than demonstrated through lesion-resolved temporal and perturbational studies (27, 28, preprint).

Accordingly, a granuloma may function simultaneously as:

- A containment structure that restricts bacterial expansion (1, 29). Containment can be assessed in human clinical studies and animal models, but its mechanistic directionality across individual human lesions requires longitudinal and, where feasible, perturbational validation. Extracellular nucleotide signaling is a plausible upstream coordination layer that may influence whether necrotic/tissue-damaging inflammatory outputs promote bacterial escape versus non-lytic containment; for example, CD39-mediated regulation of P2X7-dependent necrotic lesions supports this upstream-link concept (5).
- A permissive reservoir that supports bacterial persistence (1, 29). In human cohorts, lesion-resolved persistence dynamics remain difficult to establish definitively without matched longitudinal microbiological and imaging measurements. Mechanistically, conditions that shift inflammatory outputs toward tissue-destructive necrosis may increase the probability of bacterial release/transfer and therefore favor permissive trajectories; this is consistent with evidence that modulating CD39–P2X7 regulatory control can impact lung necrotic lesion progression (5).
- An inflammatory amplifier that sustains pathogenic signaling and tissue injury, as demonstrated in mechanistic mouse models (30). In humans, lesion-level inflammatory amplification is generally inferred from cross-sectional tissue or systemic data and is rarely tracked longitudinally within the same lesion.
- A pharmacological barrier that limits effective local drug exposure and target engagement, as demonstrated by pharmacokinetic variability across granuloma compartments in animal models (8, 29). In routine human clinical practice, paired lesion-resolved pharmacokinetic and pharmacodynamic measurements remain limited.

Clinical immunological studies further indicate that lesion trajectory is not determined solely by the magnitude of systemic immune activation (4). Systemic inflammatory measurements may correlate with overall disease state, but they cannot specify which lesion compartments receive immune pressure, whether the pressure is functionally effective, or whether local responses promote bacterial containment or tissue injury.

Human spatial and transcriptional profiling instead suggests that lesion behavior may depend on the localization of key cell populations, the interfaces at which they occur, and the local gradients that influence the probability and functional consequences of cell–cell interactions (3, 11). These data are important but are primarily associative and generally indicate cellular capacity or pathway-relevant activity rather than mechanism-specific execution. Demonstrating directionality in humans would require temporal linkage, functional assays, selective perturbation, or tightly matched longitudinal sampling, which are not routinely available in lesion-based studies.

Interpretation is further complicated by sampling bias. Human granuloma studies commonly rely on resection or biopsy specimens from advanced, drug-resistant, or treatment-refractory disease and may therefore overrepresent late-stage destructive lesions. Early restrictive-stage lesions and lesions associated with successful treatment are sampled less frequently and rarely followed longitudinally at the tissue level. Cross-system inference should consequently be made cautiously and should, where possible, triangulate evidence from human tissue with relevant models, including nonhuman primates and susceptible mouse strains (6).

### 2.2 Spatial organization generates recurrent but non-fixed microenvironments

Comparative pathology and longitudinal studies in animal models indicate that granulomas exhibit structural compartmentalization and recurrent microenvironments (6). In humans, however, the reproducibility and temporal stability of these compartments are supported mainly by cross-sectional spatial datasets and pathological observations rather than by prospective longitudinal mapping of the same lesions.

Human spatial studies and pathological reviews describe recurring microanatomical features, including macrophage-rich regions, lymphocyte-associated cuffs, necrotic or caseous areas, fibrotic capsules, neutrophil-rich regions, tertiary lymphoid structure-like aggregates, and vascular- or airway-associated niches (11, 31). Together, these features form a tissue-level organization that may influence both host defense and pathology. Nevertheless, the presence of a recurring compartment does not establish its functional connectivity, nor does it guarantee that the same cellular interactions persist over time. In particular, human datasets rarely resolve which cells interact, whether such interactions are sustained, or whether they produce effective antimicrobial activity over time (11, 31).

The composition, spatial ordering, and relative prominence of these compartments can vary according to host species, immunobiology, lesion stage, treatment status, and sampling method (6, 31). Human resection and biopsy cohorts are also not uniformly distributed across disease stages. Estimates of compartment frequency or prominence may therefore reflect sampling composition as well as genuine biological variation.

These recurrent compartments are hypothesized to regulate several processes:

- Cellular contact and interaction: Human spatial and transcriptomic studies suggest that compartmental organization influences the physical probability that immune cells encounter one another (28, preprint; 11). Physical proximity, however, is not equivalent to productive interaction. Demonstrating functional contact requires appropriate cellular, molecular, and execution-level readouts.
- Mediator diffusion: Animal models implicate granuloma architecture in controlling the diffusion and local concentration of soluble mediators (8). Whether the same gradients occur with comparable magnitude and functional consequences in matched human lesions remains insufficiently characterized.
- Antibiotic and nutrient access: Experimental models show that lesion compartments can constrain access to antibiotics and nutrients (8). In human lesions, nutrient availability is rarely measured directly, and local antibiotic exposure is often inferred from imaging or pharmacokinetic models rather than directly quantified.
- Microenvironmental signaling: Human immunometabolic mapping and *in vitro* studies indicate that local compartments can differ in oxygen tension, lipid and metabolite availability, and ECM stiffness (27, 28, preprint). Direct causal links between these cues and mechanism-specific cell-death execution in human tissue remain incompletely established.

Spatial organization may therefore create recurrent opportunities or constraints for immune function, but it should not be interpreted as a deterministic anatomical program. Spatial-proximity models and immunological studies emphasize that co-localization is often necessary for productive immunity but is not sufficient to establish it (32, preprint; 33). In clinical datasets, sustained productive connectivity that predicts lesion outcome is rarely demonstrated directly.

Mechanistic and nonhuman-primate studies further indicate that the presence of immune cells does not guarantee effective antimicrobial activity. Physical and biochemical barriers may disrupt sustained antigen presentation, coordinated myeloid activation, or antimicrobial execution within the compartments in which bacteria are located (32, preprint; 33). Directly tracking antigen presentation, cellular interaction, and execution-level antimicrobial events within the same lesion compartment over time remains a major limitation of human research.

Accordingly, spatial co-localization should be treated as an empirical observation requiring validation rather than as evidence of causality or directionality. Establishing causal direction requires temporal designs, functional measurements, selective perturbation, or rescue experiments that are generally unavailable in observational human tissue studies.

### 2.3 Restrictive and permissive lesion states: dynamic operational poles

Clinical, immunological, and imaging studies in cynomolgus macaques support conceptualizing lesion outcomes along a continuum rather than as a binary active/inactive dichotomy (**Figure 1**) (7). This continuum model is informed by longitudinal animal studies and cross-sectional observations in humans, but prospective longitudinal mapping of lesion trajectories in humans remains limited.

**Figure 1:**
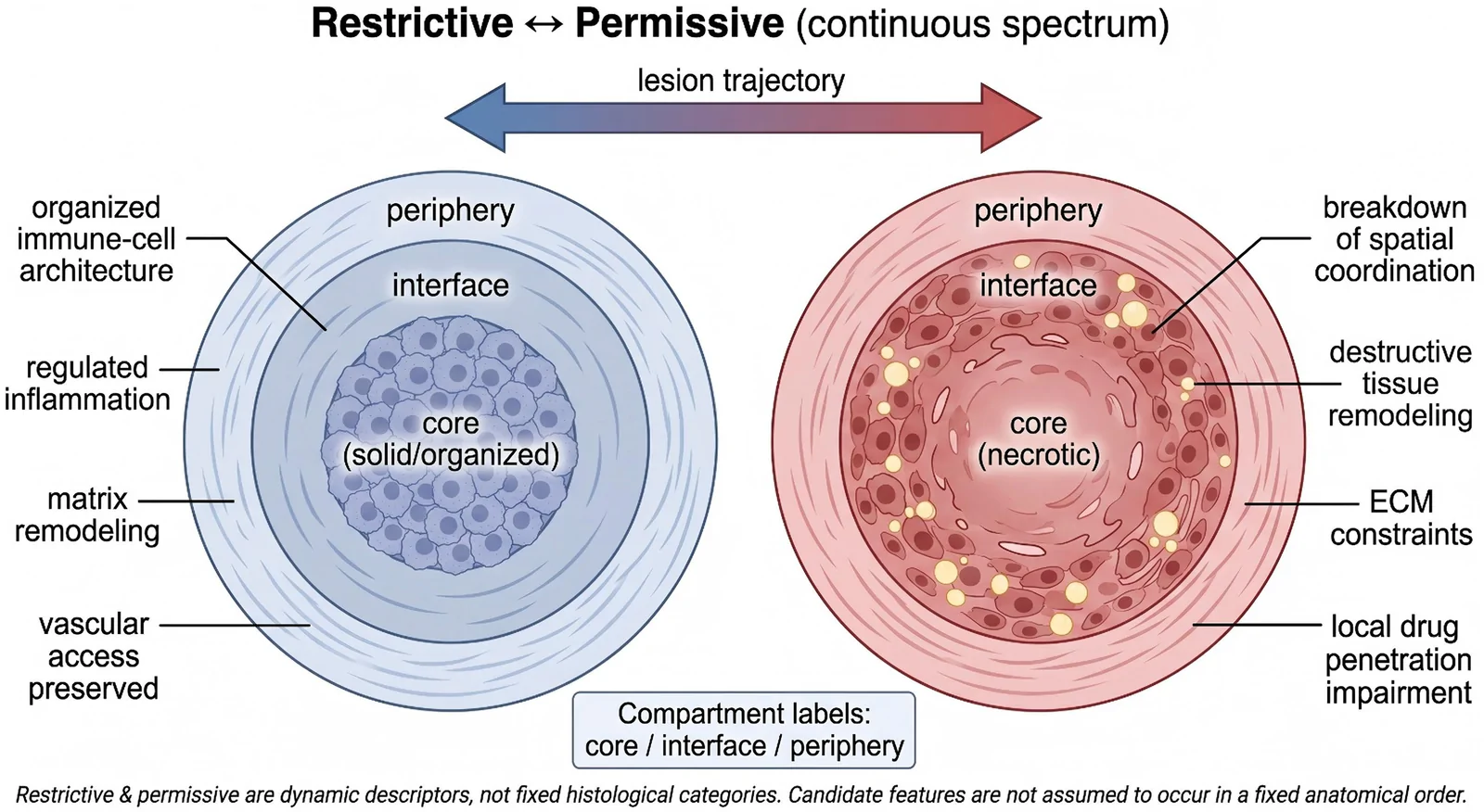
Tuberculosis lesions occupy dynamic restrictive-to-permissive trajectories. TB lesions within the same host may differ in bacterial burden, tissue architecture, inflammatory organization, necrosis, cavitation, and local drug exposure. “Restrictive” and “permissive” are used as dynamic descriptors of lesion behavior rather than fixed histological categories. The figure illustrates candidate factors associated with lesion trajectory, including immune-cell organization, matrix remodeling, vascular access, metabolic context, and antimicrobial penetration. These features are not assumed to occur in a fixed anatomical order.

In this Review, restrictive and permissive are used as operational descriptors of lesion behavior rather than fixed histopathological categories. Whenever possible, their classification should be based on longitudinal microbiological, imaging, pathological, and, where relevant, pharmacological measurements (6, 7). In human patients, true lesion-level longitudinal sampling is uncommon and may be confounded by treatment timing, lesion accessibility, and the inability to obtain repeated tissue samples from the same anatomical site.

#### 2.3.1 Restrictive lesion behavior

Restrictive behavior refers to a dynamic state characterized by preserved or partially preserved lesion organization, limited bacterial expansion, and limited destructive remodeling. Productive cellular coordination may contribute to this state, but “productive connectivity” should not be inferred from cellular proximity alone and is rarely measured at the execution level in human subjects (26, 34).

Restrictive behavior should be supported, where possible, by some combination of the following:

- Bacterial control over time: stable or decreasing bacterial burden across longitudinal measurements. This is well established as an operational outcome in animal microbiology, but matching culture or quantitative PCR data to specific lesion compartments is uncommon in humans.
- Limited destructive remodeling: limited progression of necrotic or caseous core volume, stabilization of cavitation-prone architecture, and/or stable or improving fibrosis-related imaging or pathological indices over repeated assessments. Such longitudinal measurements are more feasible in animal models than in human lesions, where serial tissue sampling is rarely possible.
- Sustained local functional engagement: evidence that local immune programs remain coordinated and retain antimicrobial or containment-associated activity. Human lesion-resolved spatial and transcriptional signatures can support this inference, but they do not establish execution or prove that systemic immune signals are effectively translated into local control (3).
- Adequate local pharmacological exposure, where relevant: evidence that an antimicrobial or adjunctive therapy reaches the lesion compartment at an exposure sufficient to support target engagement and prevent or slow deterioration. Granuloma heterogeneity in animal pharmacokinetic models demonstrates the relevance of this variable, whereas lesion-level exposure and pharmacodynamic response are rarely measured together in humans (8).

These features should be treated as convergent indicators rather than as mandatory criteria. A lesion may show bacterial control despite persistent inflammation, or structural stability despite incomplete microbiological information. Such discordance should be recorded explicitly rather than resolved by assigning an unqualified restrictive label.

#### 2.3.2 Permissive lesion behavior

Permissive behavior refers to a dynamic state in which bacterial persistence or expansion is accompanied by progressive tissue injury, loss of structural containment, or other evidence of destructive remodeling. Human spatial transcriptomic studies and *in vitro* macrophage models have associated permissive phenotypes with impaired protective coordination and severe microenvironmental or metabolic gradients, including hypoxia, caseous necrosis, lipid-rich foamy macrophage niches, and progressive ECM deposition (10, 35). These associations support biological plausibility but do not establish that the gradients cause lesion progression in humans.

Permissive behavior may be supported, where possible, by some combination of the following:

- Persistence with progression: sustained or increasing bacterial burden together with worsening lesion destruction. Although this relationship can be evaluated using longitudinal microbiology and imaging in animal models, matched measurements of progressive cavitation and compartment-specific bacterial burden are rarely obtained in humans.
- Advancing structural damage: increasing necrotic or caseous volume, progression toward cavitation, or worsening matrix-injury indicators over time. Serial pathological assessment of the same human lesion is uncommon, so imaging and biomarker measures should be interpreted with attention to their resolution and specificity.
- Inflammation–containment decoupling: persistent or increasing inflammatory activity despite inadequate bacterial control. Mechanistic mouse studies support this form of decoupling (30, 36), but confirming it in human lesions requires matched temporal measurements of local inflammation and bacterial control.
- Impaired local drug exposure, where assessed: compartment-restricted drug availability or target exposure that plausibly permits bacterial survival and destructive remodeling. This variable is well documented in animal studies of granuloma pharmacokinetic heterogeneity (8), whereas direct compartment-level drug concentration and pharmacodynamic measurements remain severely limited in humans.

As with restrictive behavior, these indicators are not individually definitive. Permissive classification should be based on convergent longitudinal evidence and should distinguish bacterial persistence, structural injury, inflammatory activity, and pharmacological exposure rather than treating any single marker as sufficient.

#### 2.3.3 Operational requirements and interpretation

Because restrictive and permissive states are dynamic, classification should ideally be anchored to measurable changes over time using longitudinal microbiology, imaging, pathology, and, where relevant, lesion-resolved pharmacology (6, 7). Macaque and other animal models support this evolutionary view, but prospective longitudinal lesion sampling is not standard clinical practice. Human cross-sectional snapshots should therefore be described as stage-associated rather than definitive trajectory assignments unless temporal validation is available (1, 6).

The restrictive–permissive spectrum should also not be used circularly. Neither trajectory is assumed to be automatically explained by a predefined Necro-Mosaic axis or spatial module (1, 7). Instead, these lesion behaviors are treated as outcomes that the F-, P-, and N-axis hypotheses must explain mechanistically. The relevant question is whether axis-associated observations demonstrate pathway capacity, pathway engagement, mechanism-specific execution, or causal contribution, and what findings would falsify the proposed explanation.

Thus, the lesion spectrum defines the phenotypic and pathological outcomes that require explanation, whereas the mechanistic axes and their spatial or temporal relationships remain empirical. Current disconfirmatory causal tests for lesion-specific axis contributions are largely restricted to preclinical systems (3, 29, 33). **Table 1** summarizes the operational features of restrictive, permissive, and indeterminate or mixed lesion trajectories; **Table 2** summarizes the evidence thresholds required to interpret the candidate mechanistic axes. A lesion that cannot be classified confidently because of discordant measurements, inadequate temporal information, or insufficient execution-level evidence should be designated indeterminate or mixed, rather than forced into a restrictive or permissive category.

**Table 1:** Operational framework for restrictive, permissive, and indeterminate/mixed lesion trajectories.

Feature domain	Restrictive trajectory	Permissive trajectory	Indeterminate/mixed trajectory	Measurement approach	Evidence status
Lesion architecture	Relatively preserved organization; limited destructive progression	Necrotic expansion, cavity formation, tissue destruction, or progressive remodeling	Inconsistent or mixed structural signals; attribution to a single trajectory cannot be confidently assigned	CT/PET-CT; pathology; longitudinal lesion measurements	Imaging and pathology are established; execution-level molecular interpretation remains emerging and often resolution-limited
Bacterial control	Stable or declining burden under effective therapy	Persistent burden, delayed sterilization, or relapse-associated persistence	Burden dynamics vary across lesions and compartments; trajectory-level assignment cannot be reliably inferred from systemic outcomes alone	Culture, molecular bacterial load, lesion-specific measures where available	Established for systemic microbiological outcomes; lesion-level attribution is limited in cross-sectional or spot-based readouts
Immune organization	Cellular interactions compatible with local containment and regulated inflammation	Dysregulated or spatially disconnected inflammatory and myeloid responses	Coexistence of containment-compatible and damage-associated neighborhoods; mixed cellular composition and spatial disconnection may co-occur	Multiplex imaging; spatial transcriptomics/proteomics; tissue histology	Emerging; mixed and indeterminate profiles must be accommodated within a non-exclusive-axis framework, and their frequency remains an empirical question
Metabolic and microenvironmental context	No single defining metabolic profile	May include altered lipid metabolism, redox imbalance, vascular limitation, and ECM remodeling	Compartment-dependent metabolic signatures with uncertainty due to sampling resolution and lesion-stage variability	Spatial metabolomics; hypoxia probes; lipid imaging; histology	Emerging and context dependent; interpretation requires lesion-resolved measurements and temporal linkage
Drug exposure	Adequate exposure in relevant lesion regions	Heterogeneous penetration or suboptimal local exposure	Uneven exposure across compartments; effective exposure may be present in some regions but absent in others	MALDI-MSI; lesion PK/PD modeling; drug quantification	Established principle; often unavailable at sufficient lesion-resolved resolution in routine studies
Cell-death-associated processes	No prespecified protective pattern	Candidate F/P/N-axis-associated processes may be present singly or in combination	Multiple candidate processes may coexist or be variably detected; execution-level evidence is insufficient for single-axis attribution	Execution-level spatial assays and perturbation studies	Hypothesis-generating; mixed and indeterminate profiles require execution- and time-resolved validation before mechanistic labeling
Blood biomarkers	May reflect overall disease activity or treatment response	May reflect systemic inflammatory burden or aggregate lesion burden	Systemic signals reflect aggregate contributions and may be discordant with lesion-level mechanisms due to multi-lesion heterogeneity	Transcriptomics, proteomics, metabolomics	Exploratory correlates only; cannot assign lesion-level F/P/N states without lesion-resolving calibration in independent cohorts

Restrictive, permissive, and indeterminate/mixed are dynamic descriptors of lesion trajectory, not mutually exclusive patient categories. A single patient may harbor lesions with different trajectories simultaneously. Mixed and indeterminate trajectories must be accommodated within a non-exclusive-axis framework; their frequency remains an empirical question. Abbreviations: CT, computed tomography; ECM, extracellular matrix; F/P/N, F-axis-associated lipid peroxidation/P-axis-associated coordinated inflammatory cell-death activity/N-axis-associated neutrophil extracellular activity; MALDI-MSI, matrix-assisted laser desorption/ionization mass spectrometry imaging; PET, positron emission tomography; PK/PD, pharmacokinetics/pharmacodynamics.

**Table 2:** Evidence framework for non-exclusive necro-mosaic axes in TB lesions.

Mechanistic axis	Biological construct	Spatial distribution (empirical question)	Capacity/susceptibility markers	Engagement markers	Evidence threshold and permitted inference	Current TB evidence & key limitation	Evidence system/source	Priority falsifiable test
F axis (F-axis-associated lipid peroxidation)	Iron-dependent lipid peroxidation and membrane injury; ferroptosis is an established regulated cell-death pathway, but axis assignment does not itself establish ferroptosis	May occur in lipid-rich, iron- and redox-stressed lesion regions; any relationship to oxygen gradients must be measured, not presumed as a fixed licensing compartment	ACSL4, GPX4, SLC7A11; iron-handling and lipid-metabolism signatures	Lipid ROS, oxidized phospholipids, altered antioxidant activity	Human tissue: orthogonal evidence consistent with ferroptosis-associated lipid-peroxidation injury/engagement; no single lipid-oxidation marker establishes ferroptosis. Mechanism-specific ferroptosis claim in a matched experimental system requires iron and oxidized-phospholipid dependence, altered GPX4 or related antioxidant-system function, exclusion of plausible alternatives, and pharmacological or genetic rescue.	Experimental TB studies support lipid-peroxidation-related injury biology, whereas direct lesion-resolved evidence for mechanism-specific ferroptosis in human TB remains limited. Genoula et al. (35) supports HIF-1α-associated foamy-macrophage/lipid-metabolic biology, not direct ferroptosis execution.	Amaral et al. (17): TB experimental model; Genoula et al. (35): macrophage/metabolic TB context; human lesion-specific ferroptosis execution: limited	In a necrotizing TB model, selectively modulate ferroptosis-associated pathways and test whether lipid-peroxidation burden, necrotic expansion, bacterial burden, and lesion trajectory change.
P axis (coordinated inflammatory cell-death activity)	Potentially coordinated crosstalk among pyroptotic, apoptotic, and necroptotic branches; axis assignment does not itself establish a PANoptotic mechanism or PANoptosome	May be enriched in inflammatory cellular neighborhoods; no specific compartment should be assumed	Upstream purinergic regulators (e.g., P2RX7, CD39); ZBP1, RIPK3, CASP8, GSDMD; inflammasome-related genes; death-receptor pathway components	Activated or cleaved CASP8, MLKL, GSDMD; caspase/inflammasome activity; DAMP release	Coordinated multi-branch activity: same-cell, multiplex protein-level evidence for activated apoptotic, necroptotic, and pyroptotic branches, with structural and cell-identity controls. PANoptotic mechanism: additionally requires a proposed common regulatory node, temporal/functional linkage, loss-of-function and rescue, and demonstration that perturbation changes coordinated activity and lesion-relevant outcomes.	TB lesion evidence supports neither PANoptosome assembly nor a causal PANoptotic mechanism. Spatial transcriptomics can show neighborhood-level co-occurrence but cannot establish same-cell coordination or integrated execution.	Christgen et al. (19)Karki et al. (20): mechanistic framework; Qiu et al. (10): TB spatial transcriptomic co-occurrence, not same-cell execution; Almeida-Santos et al. (5): upstream purinergic regulation of necrosis.	In a relevant model, perturb an upstream convergence node (e.g., the CD39–ATP-P2X7 axis) and determine whether coordinated inflammatory cell-death activity and lesion outcomes improve without loss of bacterial control.
N axis (neutrophil extracellular activity)	Neutrophil extracellular activity, including NET-associated molecular signals, structurally defined extracellular chromatin–granule-protein material, and extracellular-effector persistence; these are distinct from neutrophil abundance and NETotic cell death	May occur in measured cavity-associated, airway-facing, necrosis-adjacent, ECM-remodeling, or other neutrophil-accessible regions; no location is definitional. Lesion-resolved NET-structure localization in human TB remains incompletely resolved.	Neutrophil abundance; PADI4, ELANE, MPO, CYBB transcripts or proteins	Histone citrullination, extracellular DNA, granule-protein redistribution, extracellular protease activity; each requires structural and compartmental interpretation	NET-associated extracellular structures: extracellular DNA structurally co-localized with CitH3 and relevant neutrophil proteins, with compartmental, antibody, and non-NET extracellular-DNA controls. NETotic cell death requires additional lytic, temporal, and functional evidence. DNase-mediated extracellular-DNA degradation and efferocytosis should be assessed separately; clearance competence is currently an unvalidated contextual covariate.	Human TB evidence supports cavity-associated neutrophilic inflammation, NET-related material in respiratory samples, and MMP-8-associated matrix-destructive biology. Recent resection-based human evidence links NETosis-associated markers with tissue destruction, but because the design is cross-sectional, and because marker specificity and structural definitions may be limiting, the evidence does not establish (i) NETotic cell death execution as such, nor (ii) lesion-trajectory causality. Dallenga et al. (37) addresses Mtb-induced neutrophil necrosis rather than spatial NET structures in human granulomas.	Ong et al. (38): human pulmonary TB/respiratory samples and tissue-associated neutrophil biology; Fisher et al. (21): cross-sectional human resection tissue; Dallenga et al. (37): experimental neutrophil-necrosis study; Farrera and Fadeel (22): non-TB/general NET-clearance mechanism	In a longitudinal necrotizing or cavitary model, selectively modulate a defined N-axis component and test whether structurally defined NET-associated material, matrix injury, necrotic expansion/cavitation, bacterial burden, and dissemination change within a prespecified time window. Analyze extracellular-DNA degradation and efferocytic clearance separately.

The F, P, and N axes are non-exclusive. Their spatial distributions, overlap, temporal sequence, and relationship to lesion progression are empirical questions. Pathway-associated transcripts or proteins indicate capacity or engagement unless structurally or functionally stronger evidence is also present. Abbreviations: ACSL4, acyl-CoA synthetase long-chain family member 4; CASP8, caspase-8; CD39, cluster of differentiation 39 (ectonucleoside triphosphate diphosphohydrolase-1); CitH3, citrullinated histone H3; CYBB, (e.g., NOX2-associated) NADPH oxidase beta; DAMP, damage-associated molecular pattern; ECM, extracellular matrix; ELANE, neutrophil elastase; GPX4, glutathione peroxidase 4; GSDMD, gasdermin D; MLKL, mixed lineage kinase domain-like pseudokinase; MPO, myeloperoxidase; NET, neutrophil extracellular trap; P2RX7, purinergic receptor P2X 7; PADI4, peptidyl arginine deiminase 4; ROS, reactive oxygen species; TB, tuberculosis; ZBP1, Z-DNA-binding protein 1.

## 3 Cellular interaction circuits and lesion heterogeneity

TB lesion outcomes depend not only on immune-cell abundance or systemic inflammatory markers, but also on the quality, timing, and tissue context of cellular interaction circuits (3, 11). Granuloma architecture and local microenvironmental states can influence immune-cell access, antigen presentation, cytokine diffusion, matrix remodeling, vascular supply, and local drug exposure (8, 28, preprint). However, these features should be regarded as constraints and modulators of interaction probabilities rather than fixed anatomical rules that determine which programmed-cell-death (PCD)-associated process occurs in a given lesion region.

In this Review, restrictive and permissive states describe dynamic lesion trajectories rather than mutually exclusive biological end states. Restrictive trajectories are characterized by relatively limited bacterial growth and destructive remodeling over time, whereas permissive trajectories include persistent bacterial burden, necrotic expansion, structural deterioration, cavitation, and/or inadequate treatment response (6, 7). Individual patients may harbor lesions at different positions along this continuum simultaneously, and cross-sectional lesion observations should be interpreted as stage-associated unless longitudinal validation is available.

The Necro-Mosaic framework generates testable hypotheses about whether F-axis-associated lipid-peroxidation injury, P-axis-associated coordinated inflammatory cell-death activity, and N-axis-associated neutrophil extracellular activity contribute to these trajectories. These axes are non-exclusive mechanistic hypotheses, not cell-type labels and not predetermined spatial modules. Their relevance must be evaluated by distinguishing pathway capacity from pathway engagement and mechanism-specific execution, and by directly measuring spatial distribution, temporal order, overlap, and functional consequences. Spatial co-localization, cell-type abundance, or transcriptomic enrichment alone remains hypothesis-generating and does not establish pathway engagement, execution, or causal contribution (**Tables 2**, **3**; **Supplementary Table S1**).

**Table 3:** Technologies and minimum standards for evaluating necro-mosaic hypotheses.

Technology	Primary contribution	Appropriate inference	Critical limitation	Minimum controls/validation requirement	Representative evidence system/source
Spatial transcriptomics	Maps pathway-associated gene expression and cellular neighborhoods	Neighborhood-level co-occurrence and pathway capacity	Spot-based platforms cannot establish same-cell co-expression or executed cell death	Cell-type deconvolution/segmentation where possible; orthogonal protein or imaging validation; lesion-compartment annotation	Human lesion tissue; animal models
Single-cell RNA-seq/multi-omics	Defines cell populations and transcriptional states	Cell-specific pathway capacity; candidate cell populations	Dissociation removes spatial context and may bias fragile cell populations	Viability and dissociation controls; batch correction; orthogonal spatial validation	Human samples; animal models; ex vivo systems
Multiplex immunofluorescence/IMC/MIBI-TOF	Detects proteins and spatial relationships	Protein co-localization, cell identity, tissue neighborhood	Requires rigorously validated antibodies and appropriate controls	Positive/negative controls, isotype/antibody validation, segmentation quality control, compartmental annotation	Human lesion tissue; animal models
Lipid-peroxidation assays	Detects oxidative lipid injury	Ferroptosis-associated lipid-peroxidation injury/engagement; not mechanism-specific ferroptosis from a single assay	4-HNE, MDA, C11-BODIPY oxidation, and morphology may reflect generalized oxidative injury	Orthogonal iron/oxidized-phospholipid/antioxidant-system evidence; matched perturbation/rescue in experimental systems	Human tissue plus matched experimental systems
NET-focused imaging	Detects CitH3, extracellular DNA, and neutrophil proteins	Structurally defined NET-associated extracellular material when extracellular DNA co-localizes with CitH3 and neutrophil proteins under appropriate controls	Does not establish NETotic cell death; extracellular DNA may derive from generalized necrosis or other cell death	Compartmental controls, antibody controls, non-NET extracellular-DNA controls, structural co-localization requirements	Human lesion tissue; respiratory samples; animal models
Spatial ATAC-seq/CUT&Tag/CUT&RUN	Profiles chromatin accessibility or histone marks	Tests epigenetic susceptibility hypotheses	Direct TB granuloma evidence remains limited	Cell-type assignment; orthogonal transcription/protein validation; lesion-stage and treatment-context annotation	Emerging in human/animal granuloma studies
MALDI-MSI and lesion PK/PD methods	Maps drug, lipid, and metabolite distribution	Local drug exposure and spatial pharmacology	Drug presence does not demonstrate target engagement or efficacy	Analytical calibration, tissue registration, paired target-engagement or outcome measures	Human tissue; animal PK/PD studies
PET/CT and serial CT	Measures lesion evolution *in vivo*	Temporal change in lesion burden, cavitation, and treatment response	Cannot independently identify molecular cell-death mechanisms	Standardized timing, radiologic criteria, and linkage to tissue or molecular measurements where feasible	Clinical human studies; animal imaging
Organoids/microfluidic systems/3D granuloma models	Enables controlled perturbation of local conditions	Mechanistic testing under defined conditions	Simplified architecture and incomplete representation of human lesions	Defined composition, replicate perturbation, and benchmarking against *in vivo* readouts	*In vitro*/ex vivo models
Necrotizing animal models	Tests lesion-level causality and intervention effects	Perturbation of tissue damage, bacterial burden, and lesion trajectory	Species-specific lesion biology limits direct extrapolation to human TB	Prespecified lesion endpoints, microbiology, pathology, timing, and rescue/negative-control arms	Mouse, rabbit, nonhuman primate, or other TB models

Strong causal evidence requires integration of spatial localization, structurally or functionally stronger pathway measurements, longitudinal lesion outcomes, and selective perturbation. No individual platform is sufficient on its own. Abbreviations: ATAC-seq, assay for transposase-accessible chromatin with sequencing; CitH3, citrullinated histone H3; CUT&RUN, cleavage under targets and release using nuclease; CUT&Tag, cleavage under targets and tagmentation; IMC, imaging mass cytometry; MALDI-MSI, matrix-assisted laser desorption/ionization mass spectrometry imaging; MIBI-TOF, multiplexed ion beam imaging by time of flight; NET, neutrophil extracellular trap; PET/CT, positron emission tomography/computed tomography; PK/PD, pharmacokinetics/pharmacodynamics; RNA-seq, RNA sequencing; TB, tuberculosis. Representative sources for assay principles and limitations include Amaral et al. (17) for ferroptosis-associated lipid-peroxidation interpretation; Fisher et al. (21) for NET-associated extracellular-structure interpretation; Farrera and Fadeel (22) for general NET-clearance biology; Qiu et al. (10) and Sawyer et al. (11) for spatial-resolution limitations in TB lesions; and Prideaux et al. (9) and Cicchese et al. (8) for lesion-resolved pharmacology.

### 3.1 Macrophage–*M. tuberculosis* interactions: cellular diversity and local context

Macrophages are major cellular targets of Mycobacterium tuberculosis and central components of granuloma organization (1, 29). Macrophage populations vary in ontogeny, transcriptional state, lipid metabolism, redox balance, antimicrobial capacity, antigen-presentation competence, and interactions with lymphocytes, neutrophils, stromal cells, and bacteria (13, 23, 39). This diversity is not adequately represented by a simplified M1/M2 classification, which does not capture the continuum of macrophage states observed in inflamed and infected tissues (40, 41).

Depending on local lipid availability, nutrient limitation, oxygen tension, cytokine exposure, bacterial burden, and access to antimicrobial effectors, macrophage states may contribute to bacterial containment, inflammatory signaling, tissue repair, or persistence (35, 36). These context-dependent characteristics can alter susceptibility to oxidative stress, inflammatory signaling, and regulated cell-death pathways that may map onto the F-axis (ferroptosis-associated lipid peroxidation) and P-axis (inflammatory cell-death convergence). However, they do not independently establish mechanism-specific execution (e.g., ferroptosis vs other death modalities, or PANoptosis-like coordinated execution) within a specific lesion region. 

Accordingly, the central question within Necro-Mosaic is not which death program is presumed to dominate at a specific location. Rather, it is whether defined macrophage states and local microenvironmental conditions are associated with measurable evidence of pathway capacity, pathway engagement, or mechanism-specific execution, and whether these observations precede lesion progression or occur as consequences of established tissue injury. Answering this question requires lesion-resolved measurements with structural, temporal, functional, exclusion, perturbation, and, where feasible, rescue controls (**Figure 2**; **Tables 2**, **3**; **Supplementary Table S1**).

**Figure 2:**
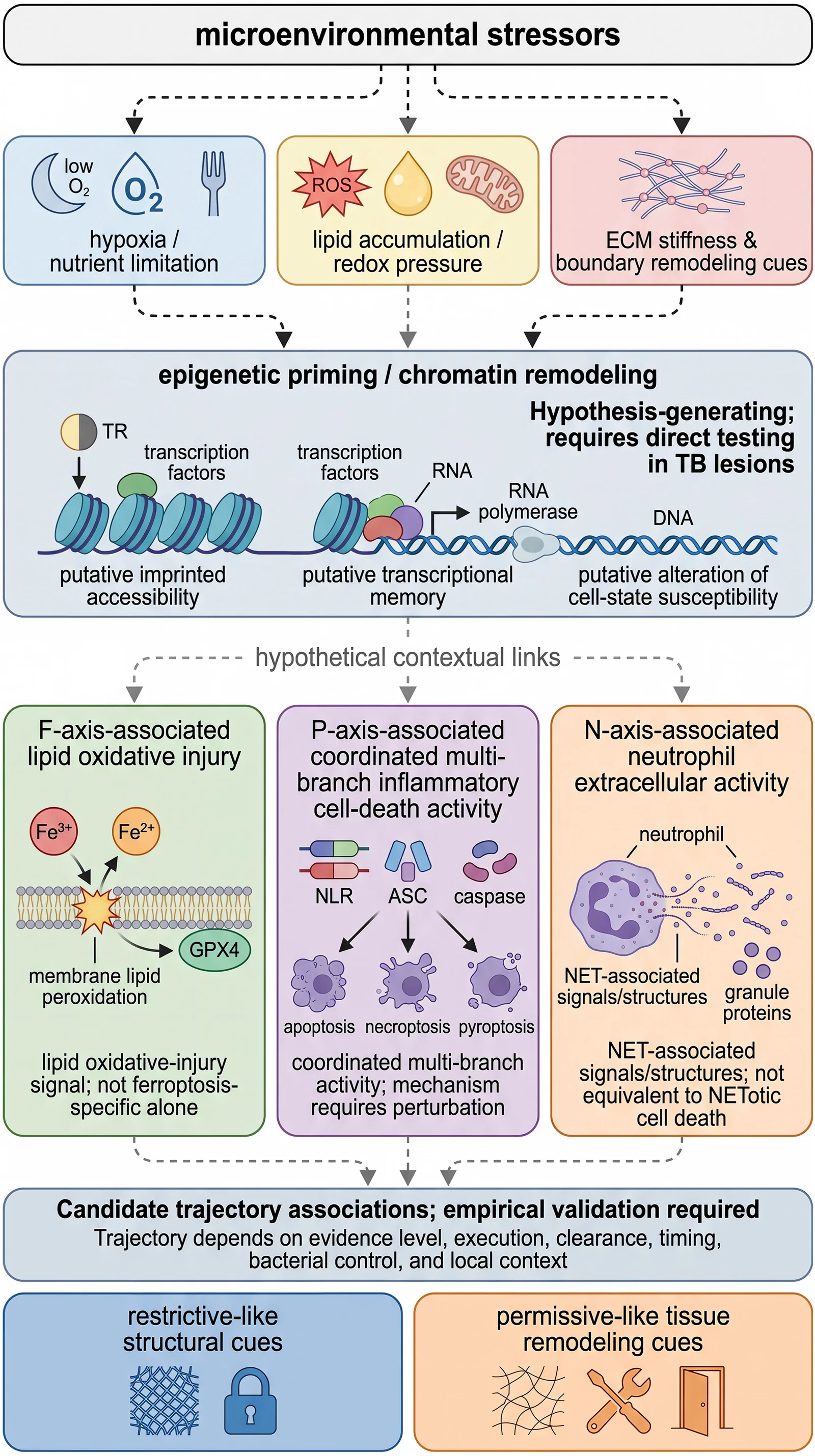
Hypothesized contextual influences on non-exclusive axis-associated processes in TB lesions. Local microenvironmental stressors—including hypoxia/nutrient limitation, lipid accumulation/redox pressure, and extracellular-matrix stiffness or boundary-remodeling cues—may influence cell state, injury susceptibility, and pathway-relevant capacity. Epigenetic priming/chromatin remodeling, including putative imprinted accessibility, transcriptional memory, and cell-state licensing, is presented as a hypothesis requiring direct testing. The F-, P-, and N-axis panels represent non-exclusive, potentially overlapping axis-associated signals rather than predefined lesion compartments or fixed mechanistic categories. Lipid-peroxidation signals do not alone establish ferroptosis; coordinated multi-branch cell-death activity does not alone establish an integrated PANoptotic mechanism; and NET-associated signals or structures do not alone establish NETotic cell death. NET-structure claims require extracellular DNA structurally co-localized with citrullinated histone H3 and neutrophil proteins, with appropriate compartmental and negative controls. Associations between any axis-associated pattern and restrictive-like or permissive-like tissue-remodeling trajectories are candidate relationships requiring empirical validation and may vary with evidence level, execution, clearance, timing, bacterial control, treatment, and local context. Direct testing will require spatial multi-omic profiling, multiplex imaging and protein assays, longitudinal sampling, and perturbation/rescue studies.

### 3.2 Macrophage–T-cell communication: necessary but spatially constrained immunity

Bidirectional communication between infected macrophages and T cells is central to adaptive immunity against TB (1, 33). Effective local control requires antigen recognition, cytokine-mediated activation of infected cells, and regulation of inflammation sufficient to limit bacterial growth without causing excessive tissue injury.

The presence of T cells within a lesion, however, does not demonstrate productive macrophage–T-cell interaction. T cells may be physically separated from infected macrophages, encounter impaired antigen presentation, be constrained by inhibitory myeloid programs, or be functionally altered by local metabolic, hypoxic, and inflammatory conditions (3, 12). Consequently, systemic immune activation, blood-based T-cell responses, or intralesional T-cell abundance should not be equated with lesion-level antimicrobial efficacy (42, 43).

Disrupted macrophage–T-cell communication could contribute to permissive lesion evolution by weakening local bacterial containment or sustaining inflammation that is not translated into effective antimicrobial action. Whether such disruption is causally linked to F-axis injury, P-axis inflammatory cell-death activity, and/or N-axis neutrophil extracellular activity remains an empirical question. Testing this hypothesis requires spatial measurements linked to longitudinal lesion outcomes and, where feasible, selective perturbation studies that establish directionality (**Tables 2**, **3**; **Supplementary Table S1**).

### 3.3 Neutrophils and suppressive myeloid states

Neutrophil functions in TB are context dependent. Early recruitment may contribute to inflammatory signaling and antimicrobial responses, whereas persistent neutrophil-rich inflammation has been associated with severe disease, necrosis, and cavitation in specific models and clinical cohorts (37, 44). Neutrophils can release proteases, reactive oxygen species, cytokines, and extracellular chromatin-containing structures, each of which may influence tissue integrity and local immune responses. In particular, neutrophil-derived matrix metalloproteinases, including MMP-8, and other granule proteases have been implicated in extracellular-matrix destruction and cavitary progression (38, 44).

Neutrophil abundance should not be interpreted as evidence of NET formation. Similarly, expression of PADI4, ELANE, MPO, or other neutrophil-associated genes or proteins may indicate neutrophil identity or pathway-relevant capacity but does not demonstrate persistent NET-associated structures, NET release, or NET-associated cell death at a lesion interface. Direct *in situ* evidence for NET-associated extracellular structures—operationally supported by Fisher et al. (21) in human TB lung tissue—requires extracellular DNA structurally co-localized with relevant neutrophil proteins (e.g., MPO or neutrophil elastase), together with appropriate tissue-compartment, cell-integrity, and negative controls. This criterion supports NET-structure assignment rather than NETotic cell death or lesion-trajectory causality (21). Even then, such evidence should be interpreted as NET-associated extracellular activity unless temporal and lytic-cell-death evidence supports a specific inference of NET-associated cell death (**Table 2**; **Supplementary Table S1**).

In parallel, suppressive myeloid programs—including inhibitory ligands, metabolic enzymes, and anti-inflammatory mediators—may contribute to impaired local immune function (3, 45). Their biological relevance is likely to depend on lesion stage, bacterial burden, cellular composition, and spatial context. These programs should therefore be evaluated as local correlates or candidate modulators of lesion biology. Their relationship to Necro-Mosaic axes must be determined through evidence tiering and temporal ordering rather than assumed from marker expression alone.

### 3.4 B cells and tertiary lymphoid structure-like aggregates

B cells and tertiary lymphoid structure (TLS)-like aggregates may reflect organized local immune responses in TB lesions (31). Depending on their maturity, cellular composition, anatomical location, and interactions with myeloid and T-cell populations, these structures may support local antigen presentation and adaptive immune coordination, or contribute to persistent inflammatory signaling (46–49).

Current evidence does not support assigning TLS-like structures a uniformly protective or pathogenic role. Their association with restrictive or permissive trajectories, bacterial control, tissue injury, and treatment response should be evaluated in longitudinal, spatially resolved studies. Relevant analyses should distinguish the presence of a lymphoid aggregate from its cellular composition, maturation state, functional activity, and relationship to bacterial localization and tissue damage.

Likewise, it is not established whether TLS-associated organization directly modulates PCD-associated processes or primarily coexists with other lesion features that influence outcome. Any proposed relationship to the F-, P-, or N-axis should therefore be assessed using the same capacity–engagement–execution logic applied elsewhere in this Review (**Tables 2**, **3**; **Supplementary Table S1**).

### 3.5 Stromal and mesenchymal networks

Stromal and mesenchymal cells—including fibroblasts, endothelial cells, pericytes, epithelial cells, and mesenchymal stromal cells—contribute to ECM deposition, tissue repair, vascular organization, immune-cell trafficking, and potentially local drug distribution (50, 51). These functions may preserve tissue integrity in some contexts while limiting immune-cell access, altering diffusion barriers, or modifying local drug exposure in others (8, 52, 53).

ECM remodeling and vascular heterogeneity may influence lesion evolution by altering physical access, nutrient and oxygen gradients, mechanical signaling, and antimicrobial exposure. However, current evidence does not establish that any specific stromal feature determines which PCD-associated process occurs within a lesion region. Stromal signals should therefore be treated as candidate modulators of permissive evolution and of the local conditions under which F-, P-, or N-axis processes may become engaged or executed (**Tables 2**, **3**; **Supplementary Table S1**).

Future studies should integrate stromal mapping with direct measures of bacterial localization, lesion architecture, microenvironmental state, drug exposure, and mechanism-specific cell-death execution. Such multimodal designs are necessary to distinguish whether stromal remodeling is an upstream regulator of tissue injury, a downstream response to inflammation, a parallel contributor, or a combination of these possibilities across lesions and over time.

#### 3.5.1 Section 3 summary

- Lesion trajectory is shaped by cellular interaction circuits, tissue architecture, bacterial burden, matrix remodeling, and local pharmacology—not by immune-cell abundance alone.
- Macrophage, T-cell, neutrophil, lymphoid, and stromal states may contribute to lesion heterogeneity, but their roles must be evaluated in context using evidence thresholds for pathway capacity, pathway engagement, and mechanism-specific execution.
- Necro-Mosaic axes are not assigned to predetermined cell types or fixed lesion regions; spatial association remains hypothesis-generating unless supported by appropriate functional, temporal, and perturbational evidence.
- Causal inference requires lesion-resolved temporal data, execution-level measurements, and, where feasible, selective perturbation and rescue experiments. Discordant or insufficient evidence should result in an indeterminate or non-interpretable assignment rather than forced classification.

## 4 Necro-mosaic: non-exclusive programmed-cell-death–associated axes in TB lesions

In TB granulomas, necrosis has often been treated as a passive end-stage. Here, we propose that necrotic patterns can be better understood as the spatially organized output of multiple PCD processes, operating under changing local constraints and enabling/limiting conditions across lesion microenvironments (54). This motivates Necro-Mosaic: a patchwork of death-associated signals in which multiple non-exclusive mechanistic axes—rather than a single uniform cell-death endpoint—may contribute to lesion heterogeneity and trajectory (**Figure 3**) (28, preprint). By design, the framework is not a compartment-to-module mapping; instead, axis-associated claims must be supported by evidence tiering (capacity → engagement → execution) at the resolution used (**Boxes 1**, **2**; **Figure 4**).

**Figure 3:**
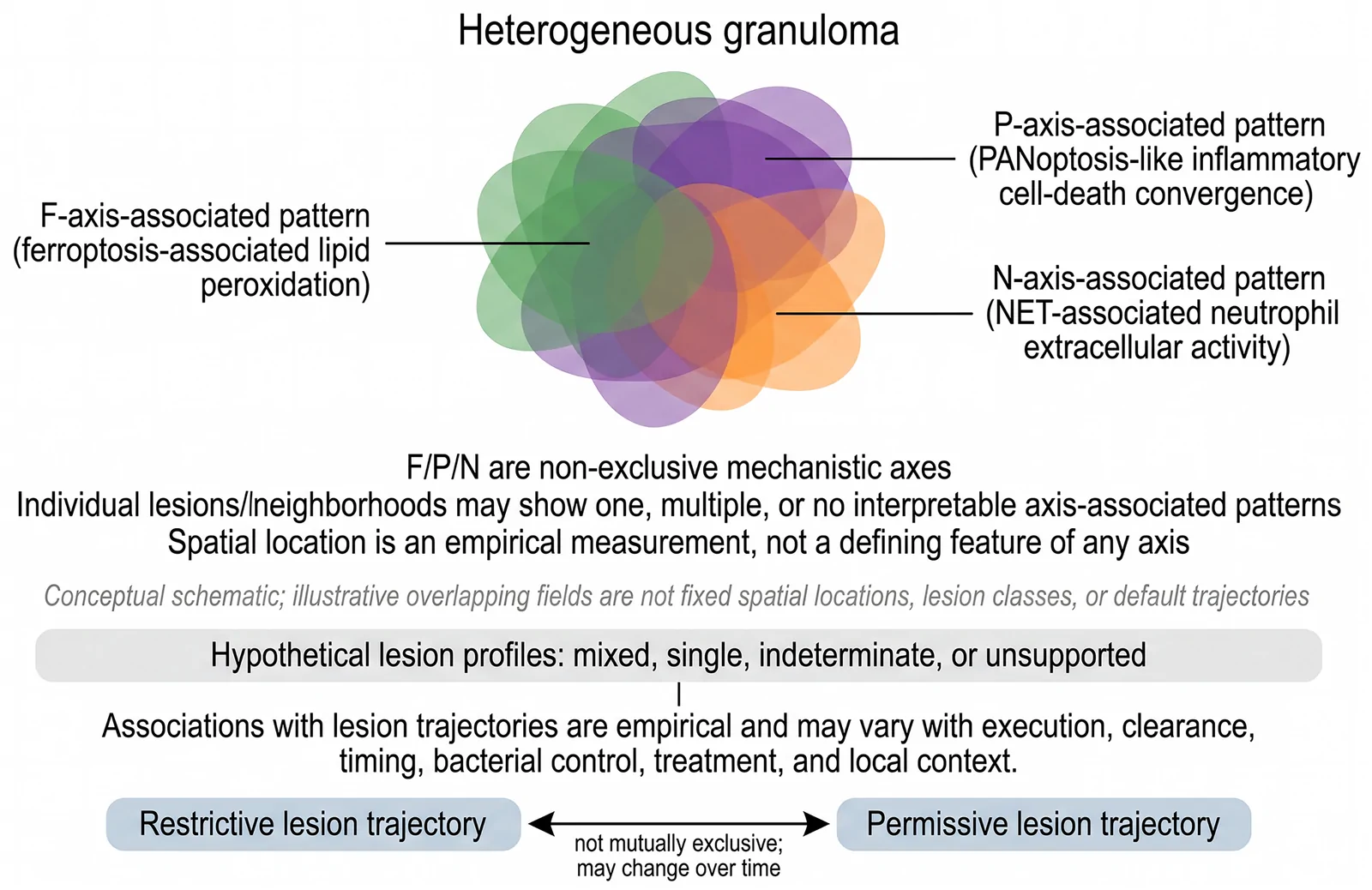
Necro-Mosaic framework: non-exclusive axis-associated patterns in heterogeneous TB lesions. The framework considers F-axis-associated lipid-peroxidation signals, P-axis-associated coordinated inflammatory cell-death activity, and N-axis-associated neutrophil extracellular activity as non-exclusive, potentially overlapping patterns. Individual lesions or local tissue neighborhoods may display single, mixed, indeterminate, or no interpretable axis-associated patterns. Spatial location is an empirical measurement rather than a defining feature of any axis, and the schematic does not imply fixed granuloma compartments, lesion classes, or default trajectories. The F-, P-, and N-axis labels denote axis-associated evidence rather than definitive proof of ferroptosis, an integrated PANoptotic mechanism, NET formation, or NETotic cell death. Associations between any axis-associated profile and restrictive or permissive lesion trajectories are candidate relationships requiring empirical validation and may vary with evidence level, execution, clearance, timing, bacterial control, treatment, and local context.

Box 1 **Operational definitions, evidentiary boundaries, and scope of the Necro-Mosaic framework.**

**Necro-Mosaic:** A hypothesis-generating framework for investigating whether multiple cell-death-associated and injury-associated processes coexist, overlap, or vary over time within and across TB lesions. It is not a validated diagnostic tool, a patient-level endotype classification, a fixed spatial taxonomy, or a treatment-selection algorithm.

**Mechanistic axis:** An analytical construct representing a biologically motivated process or process family that can be assessed independently of anatomical location. An axis is not assumed to correspond to a discrete cell type, fixed lesion compartment, or mutually exclusive mode of cell death.

**F axis:** Ferroptosis-associated susceptibility and lipid oxidative-injury biology. Evidence of lipid peroxidation alone supports oxidative lipid injury or F-axis engagement, but does not by itself establish ferroptosis execution.

**P axis:** Inflammatory cell-death convergence involving apoptosis-, pyroptosis-, and/or necroptosis-related processes. The term PANoptosis-like is used descriptively and does not imply that canonical PANoptosis has been demonstrated. Such an inference requires evidence beyond independent expression of pathway-associated genes, ideally including coordinated pathway activity, temporality, and mechanism-discriminating perturbation or rescue.

**N axis:** Neutrophil-associated effector activity, including NET-associated structures or pathways, and their potential relationship to tissue injury, microbial containment, or impaired resolution. Detection of neutrophils, extracellular DNA, citrullinated histones, or individual NET-associated markers alone does not establish NET formation, NETotic cell death, or a causal tissue-level role.

**Spatial observation:** The measured location, cellular neighborhood, microenvironmental context, or lesion state in which evidence associated with an axis is detected. Spatial location is not part of the axis definition and should not be presumed to license, exclude, or determine activity of any axis without direct measurement.

**Lesion profile:** A descriptive summary of axis-relevant evidence observed in a lesion or defined lesion region at a specified time point. Profiles may be single-axis predominant, mixed, indeterminate, or unsupported. Mixed and indeterminate profiles are expected possibilities; their prevalence, spatial organization, temporal stability, and clinical relevance remain empirical questions.

**Evidence status:** “Established,” “emerging,” and “hypothesis-generating” describe the maturity and directness of the evidence base, rather than certainty that a specific mechanism operates in an individual lesion. Established findings are reproducible and supported by appropriate primary evidence in relevant systems; emerging findings have convergent but incomplete evidence; and hypothesis-generating claims lack direct human validation and/or adequate temporal, perturbational, rescue, or clinical-outcome support. These labels are applied alongside—not instead of—the distinction between capacity, engagement, execution, and mechanism-specific validation.

**Causal interpretation:** Assignment of an axis or lesion profile does not establish that the nominated process drives lesion trajectory, bacterial control, tissue destruction, or treatment response. Causal claims require prespecified lesion-level outcomes and, where feasible, orthogonal and region-matched evidence, selective perturbation, pharmacodynamic target engagement, appropriate controls, and rescue or falsification experiments. Failure of a selective perturbation to alter the prespecified outcome, or evidence that an alternative process accounts for the observed signal, should weaken or reject the corresponding mechanistic interpretation.

Box 2 **Evidence-to-test blueprint for Necro-Mosaic axes.**

For each proposed axis, evidence should be evaluated using the following sequence.

**Observed:** What has been directly measured? Specify species/model; tissue source/disease stage; assay resolution; anatomical context; and whether the observation reflects cell abundance, pathway capacity, pathway engagement, or executed death.

**Inferred mechanism:** What causal interpretation is being proposed (driver, amplifier, compensatory response, or epiphenomenon)?

**Missing evidence:** What evidence is required before the interpretation can be strengthened?

**Falsifiable test:** What result would disconfirm the proposed mechanism?

**Figure 4:**
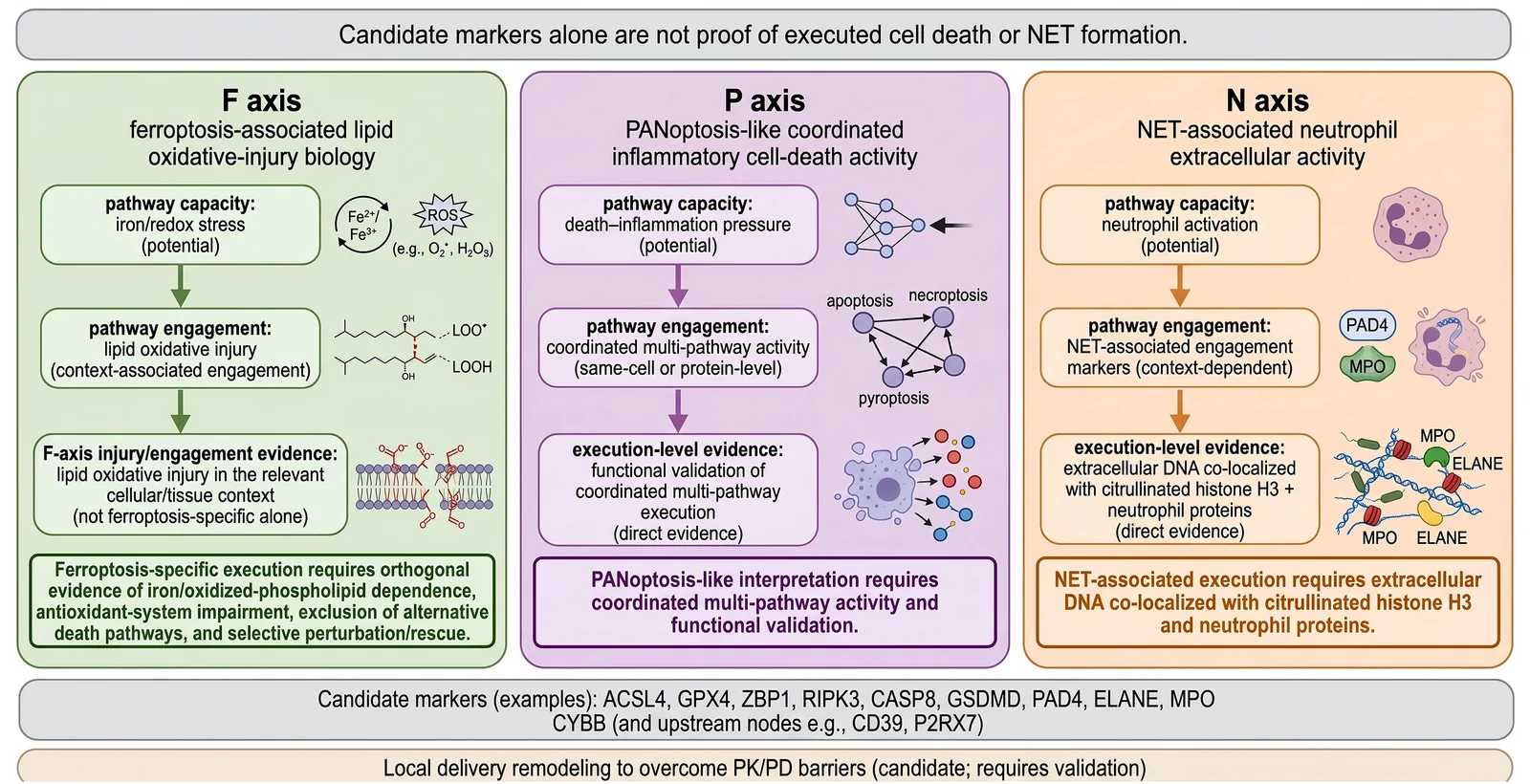
Evidence hierarchy for interpretation of F-, P-, and N-axis-associated signals in TB lesions. The framework distinguishes pathway capacity, pathway engagement, and requirements for mechanism-specific validation. Capacity and engagement indicate biological potential or axis-associated signals, but do not alone establish execution of a specific mechanism. F-axis claims require orthogonal evidence of iron- and lipid-peroxidation dependence, appropriate perturbation/rescue, and consideration of alternative death pathways. P-axis claims require temporal and functional evidence of coordinated multi-branch activity, including perturbation of a proposed common regulatory node (e.g., the CD39–ATP-P2X7 axis). For the N axis, neutrophil localization, NET-associated signals, NET structures, extracellular-effector persistence, tissue injury, and NETotic cell death are distinct claims. Extracellular DNA co-localized with citrullinated histone H3 and neutrophil proteins supports NET-structure evidence, whereas NETotic cell death requires additional lytic, temporal, and functional evidence. Candidate markers (ACSL4, GPX4, ZBP1, RIPK3, CASP8, GSDMD, PADI4, ELANE, MPO**, and CYBB**) support assay prioritization but do not alone, or by co-occurrence alone, prove ferroptosis, a PANoptotic mechanism, NET formation, or NETotic cell death.

Crucially, Necro-Mosaic is not intended to replace clinical or histopathological categories. It reinterprets necrotic architecture as structured mechanistic information: different patients—and even different lesions within the same patient—may show divergent outcomes despite similar levels of systemic immune activation, because axis-relevant evidence can differ across lesion regions, interfaces, timing, and local clearance/structural context (11). Necro-Mosaic therefore connects the where (lesion region/interface), the how (local constraints shaping whether an axis can be engaged/executed), and the what (which axis-associated processes show measurable evidence) into a framework for hypothesis-driven lesion-trajectory prediction.

Mechanistically, Necro-Mosaic relates death-associated processes to restrictive versus permissive lesion trajectories by specifying that axes may contribute to either (i) damage that is more readily resolved and remodeling that is more likely to support containment, or (ii) amplification-prone damage that sustains hostile microenvironments and permissive progression (26). Any protective versus harmful attribution cannot be assumed from necrosis alone; it requires execution-level measurement, temporal evidence, and—where feasible—selective perturbation (**Box 2**). Any potential relevance to HDT should therefore be framed as lesion-aware mechanistic stratification that still requires prospective validation, rather than an already-validated therapy-assignment instruction set (16, 24) (**Figure 4**).

### 4.1 Necro-mosaic concept: from passive necrosis to a spatially heterogeneous axis output

Classic models of granuloma necrosis as passive liquefaction struggle to explain (i) patterned necrotic architecture, (ii) distinct necrotic morphologies across lesions, and (iii) divergent outcomes under similar systemic immune activation. Necro-Mosaic reframes necrosis as an emergent lesion feature arising from the intersection of: (i) local tissue context, (ii) immune-access constraints and clearance competence, and (iii) whether specific PCD-associated axes show measurable capacity, engagement, and/or execution in a given lesion region.

A systematic mapping of axis-associated evidence to lesion microenvironments is therefore presented conceptually (**Table 2**), while the defining constraint is that axis identity is not defined by compartment (10). Axis execution may occur wherever local constraints permit engagement, and a canonical lesion region where an axis is often discussed does not automatically yield execution in a specific lesion (**Box 1**). Mixed, overlapping, or indeterminate lesion profiles should therefore be expected and handled explicitly (23, 55).

Unresolved mechanistic layers. This section does not present niche-epigenetic “priming” as an established mechanism; niche-to-epigenome coupling is treated as an untested hypothesis. Although local stressors could plausibly reshape chromatin state and alter the probability of engaging multi-branch death circuitry (56, 57), current citations do not yet provide compartment-resolved epigenomic causal evidence in TB granulomas. Accordingly, “epigenetic licensing” language is limited to hypothesis motivation for future spatial multi-omic studies.

Restrictive vs permissive outcomes are not defined by axis abundance alone. Axis-associated damage can be coupled to either productive resolution and controlled remodeling or to amplification-prone persistence and destructive progression, depending on execution-level features and on whether clearance and boundary maintenance are sufficient. Thus, lesion trajectory depends on how axis-associated damage is executed and whether the lesion environment can absorb, clear, or confine it—rather than on inflammatory markers or necrotic area in isolation.

To ensure terminological consistency, the scope and operational logic of Necro-Mosaic are defined in **Box 1** (below).

In this framework, causality is tested by whether selective perturbation of a candidate axis-associated process changes prespecified lesion-level outcomes, rather than by marker co-localization alone (**Box 2**).

### 4.2 F axis: ferroptosis-associated capacity, engagement, and evidence required to infer execution

The F axis denotes lesion contexts compatible with iron-dependent lipid-peroxidation stress and impaired antioxidant buffering, which may support ferroptosis-associated injury. In TB lesions, such conditions may plausibly occur at interfaces involving lipid-rich and necrosis-adjacent states (35). The framework does not assume a fixed oxygen compartment for F-axis activity, nor does it treat transcriptomic “ferroptosis signatures” as evidence of ferroptotic cell-death execution.

What is required to infer execution follows the tiered logic in **Boxes 1**, **2**: lipid oxidative injury must be interpreted in relation to the relevant lesion-region context, ideally including lesion-matched evidence of iron/oxidizable lipid dependence, antioxidant-defense impairment, exclusion of plausible alternative death mechanisms, and—where experimentally feasible—selective perturbation with appropriate rescue.

Oxygen-gradient caution. Hypoxia biology in TB is species-, model-, and lesion-stage dependent. Because hypoxia/HIF-1α signaling may suppress ferroptosis via mechanisms that can include lipid-droplet sequestration of peroxidisable substrates, oxygen should not be assumed to license ferroptosis execution. Where possible, lipid-peroxidation stress should be interpreted relative to measured oxygen or oxygen-transition state; without such measurements, oxygen licensing should not be asserted.

What is currently supported. Recent studies report ferroptosis-associated capacity-like patterns in TB contexts and suggest spatial enrichment of ferroptosis propensity in necrosis-adjacent or lipid-associated regions (e.g., 28, preprint). These support investigation of F-axis-associated stress states, but they do not establish that a lesion trajectory is driven by executed ferroptosis. Lipid-peroxidation readouts (e.g., 4-HNE, malondialdehyde, C11-BODIPY) indicate oxidative lipid injury or F-axis engagement, but are not by themselves specific evidence of ferroptosis execution.

Missing evidence and disconfirmation. F-axis causal support requires lesion-resolved and region-matched evidence across capacity, engagement, execution, and lesion-level consequence, including longitudinal linkage to trajectory; orthogonal assessment of lipid oxidative injury, iron dependence, antioxidant-defense status, and competing death pathways; and selective, compartment-relevant perturbation with prespecified rescue and lesion-resolved pharmacodynamic readouts (17, 18). Conversely, the F-axis interpretation should be weakened or rejected if ferroptosis-directed perturbation does not selectively modify lipid-peroxidation signals, if alternative injury pathways explain the findings, or if modulation does not alter prespecified lesion-level outcomes.

### 4.3 P axis: PANoptosis-like inflammatory cell-death convergence (with assay-resolution limits)

The P axis is treated as a PANoptosis-like inflammatory cell-death convergence axis (19, 20), not as a fully validated universal discrete execution complex in TB granulomas. Given spatial assay-resolution limits, P-axis claims must be framed as (i) pathway engagement/convergence evidence and (ii) execution evidence only when supported by appropriate controls and resolution.

Within Necro-Mosaic, P-axis evidence is expected in lesion regions where multi-branch inflammatory and cell-death signaling programs can be concurrently engaged (often discussed as inflammatory myeloid hubs) (54). However, spot-based spatial transcriptomics supports neighborhood-level co-occurrence rather than same-cell multi-pathway co-expression (10), and multi-branch execution complex claims (e.g., PANoptosome) require single-cell or protein-level *in situ* evidence of same-cell coordination with execution-level readouts and perturbational controls. Thus, spatial co-localization of multi-branch markers should be interpreted as convergence-compatible co-occurrence at the neighborhood/region level unless same-cell coordination is demonstrated. The P axis remains hypothesis-generating until selective disruption of upstream convergence nodes (or executing branches) shifts restrictive–permissive trajectory outcomes in lesion-resolved and clearance-aware readouts (**Box 2**). Extracellular purinergic signaling provides a highly relevant example of such an upstream coordination node. Specifically, ATP acting through the P2X7 receptor—whose local availability is governed by CD39 ectonucleotidase activity—can dictate whether inflammatory cascades converge into lytic outputs (5). Perturbing this CD39–P2X7 axis offers a mechanism-specific way to test whether dampening an upstream convergence signal limits pathological necrotic expansion and bacterial release without impairing bacterial containment.

Missing evidence and disconfirmation. The P-axis proposition would be disconfirmed if execution-level convergence markers decrease without corresponding changes in lesion trajectory, or if multi-branch signatures persist while lesion progression remains unaffected after accounting for exposure and clearance competence.

### 4.4 N axis: NET-associated extracellular effector activity at neutrophil–tissue interfaces

The N axis denotes NET-associated extracellular effector activity, including extracellular chromatin–granule-protein structures and their persistence in neutrophil-accessible tissue environments. It is not defined by a fixed anatomical compartment; “interface” refers to measured neutrophil-accessible tissue environments (cavity-associated, airway-facing, necrosis-adjacent, ECM-remodeling regions), rather than an assumed outer lesion boundary (6).

Human evidence supports rationale for testing N-axis hypotheses, including cavity-associated neutrophilic inflammation and neutrophil protease-mediated matrix-destructive biology (38). However, neutrophil localization/abundance does not establish lesion-resolved NET formation, NET persistence, NET-associated tissue injury, or NETotic cell death. Dallenga et al. (37) demonstrates M. tuberculosis–induced neutrophil necrosis and effects on bacterial growth but does not provide primary spatial evidence of NET-associated structures in human granuloma tissue. Likewise, Farrera and Fadeel (22) provides general mechanistic evidence on macrophage handling of NET material rather than TB-lesion-specific evidence. Recent human resection-based work reports NETosis-associated markers associated with TB tissue destruction (21), but cross-sectional evidence cannot determine temporal precedence, causality, or whether N-axis activity is an upstream driver versus a downstream correlate of lesion trajectory. Interpretation depends on marker specificity, structural co-localization, and appropriate compartmental and non-NET negative controls; consequently, lesion-resolved NET localization and causal contribution of NET-associated extracellular material to matrix destruction or cavitation remain incompletely resolved.

Evidence-tier constraint. Neutrophil abundance and PADI4/ELANE/MPO transcripts or proteins indicate lineage identity or pathway-relevant capacity, not NET formation. Histone citrullination, granule-protein redistribution, or extracellular DNA alone are similarly insufficient. Evidence for NET-associated extracellular structures in human tissue requires extracellular DNA structurally co-localized with citrullinated histone H3 and relevant neutrophil proteins, with appropriate tissue-compartment, antibody, and non-NET extracellular-DNA controls. Such evidence supports NET-structure assignment, not NETotic cell death; NETotic claims require additional lytic, temporal, and functional evidence. Clearance competence is retained only as a contextual covariate (not an operational lesion metric). DNase-mediated extracellular-DNA degradation and efferocytic removal are biologically distinct and should be measured/interpreted separately; their relevance to persistence of NET-associated extracellular material remains to be established.

Missing evidence and disconfirmation. A decisive causal test requires lesion-resolved, longitudinal perturbation in a relevant necrotizing/cavitary model. It should demonstrate selective reduction of structurally defined NET-associated extracellular material (or a defined extracellular effector), absence of compensatory alternative injury pathways, prespecified changes in matrix injury/necrotic expansion/cavitation/lesion trajectory, and preserved or improved bacterial control. Failure to improve outcomes via N-axis modulation, or worsened bacterial control alongside improved tissue injury, would argue against a simple uniformly pathogenic interpretation.

### 4.5 Additional PCD modules as upstream-to-downstream extensions (without undermining F/P/N non-exclusivity)

Other death or turnover-associated programs may contribute to lesion organization or resolution upstream or downstream of F-, P-, and N-axis features (e.g., apoptosis-like turnover supporting immunologically “silent” clearance; metabolism-linked modalities shaping fate decisions) (58). However, Necro-Mosaic does not expand into an open-ended taxonomy of PCD types.

Instead:

- the F, P, and N axes are treated as non-exclusive mechanistic axes whose relevance must be established via evidence tiering and execution-level readouts;
- additional programs are discussed only as contextual modulators of engagement/execution likelihood and clearance/resolution competence; and
- they should not be used to reintroduce fixed compartment taxonomies or to claim additional endotype categories without meeting the same falsifiability and operationalization standards (**Box 2**).

Accordingly, the value of upstream/downstream extensions is to strengthen mechanistic explanations linking microenvironmental constraints and cellular interaction circuits to lesion-trajectory outcomes—while preserving the central discipline that axis-specific claims must be separable into capacity/engagement/execution and remain testable with tissue-relevant temporal and perturbational evidence (**Box 2**) (29).

The F, P, and N axes were selected because they represent three distinct and potentially intersecting lesion-relevant process families—lipid oxidative injury, inflammatory lytic-death convergence, and neutrophil extracellular effector activity—each with plausible links to necrotic tissue injury, bacterial containment versus dissemination risk, matrix remodeling, and candidate intervention strategies, while still retaining important assay-specific inferential limitations. Apoptosis-like turnover, autophagy, and other death/stress programs remain relevant contextual modulators or component pathways; they are not excluded from the framework.

## 5 Translational implications: from lesion-aware hypotheses to staged validation

Necro-Mosaic is not currently a clinical classification system, patient endotype framework, biomarker score, or treatment-selection algorithm. Its translational value is to organize lesion-aware hypotheses, specify evidentiary thresholds, and define the staged studies required before F-, P-, and N-axis–associated processes can be considered therapeutic targets (**Figure 5**).

**Figure 5:**
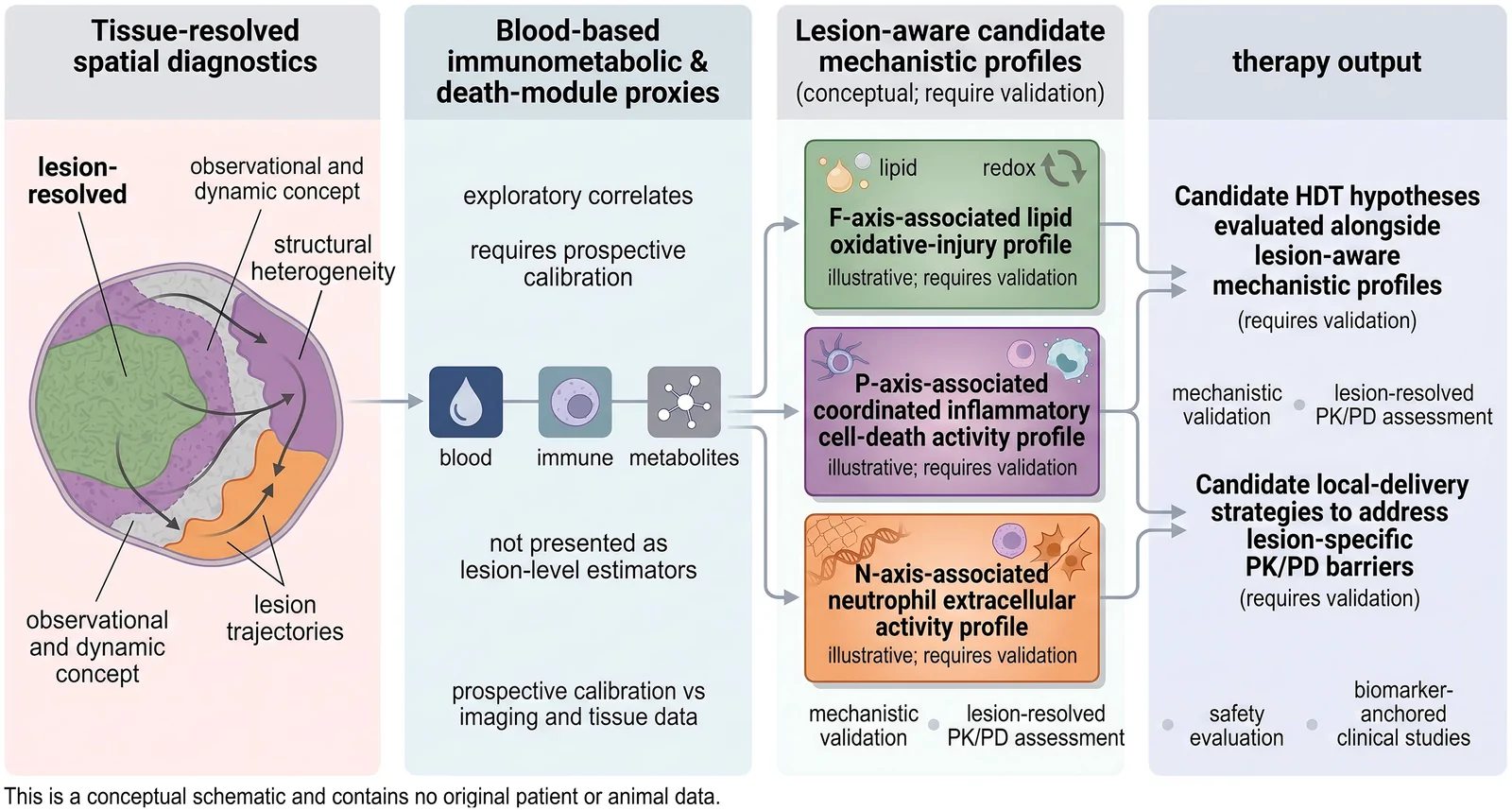
Staged translational validation of lesion-aware, axis-associated hypotheses in TB. Conceptual workflow for evaluating candidate F-axis-associated lipid oxidative-injury, P-axis-associated coordinated inflammatory cell-death activity, and N-axis-associated neutrophil extracellular-effector activity in a lesion-aware manner. Lesion-resolved spatial diagnostics identify structural heterogeneity and dynamic lesion trajectories. Blood-based immunometabolic and death-mode measures are treated as exploratory correlates that require prospective calibration against predefined lesion-resolved imaging outcomes and, where feasible, tissue-resolved reference measurements; they are not presented as lesion-level estimators. Tissue-based spatial and molecular assays, when clinically indicated and feasible, test pathway localization and the level of evidence for pathway engagement and/or mechanism-specific execution, with explicit separation of capacity, engagement, and execution. Candidate host-directed therapy hypotheses—including upstream modulators of necrotic outputs such as purinergic ATP–P2X7 signaling shaped by ectonucleotidase activity (e.g., CD39) and downstream N-axis effector targeting options involving NET-linked protease/oxidative pathways (e.g., MPO/NE and NOX2/CYBB-associated signaling)—should proceed through mechanistic validation, lesion-resolved PK/PD assessment, safety evaluation, and biomarker-anchored clinical studies. For N-axis-directed development, therapeutic plausibility requires lesion-resolved target engagement and separation of NET-associated extracellular effector persistence from generalized inflammation, with explicit monitoring of bacterial control, dissemination/secondary-infection risk, and impairment of protective antimycobacterial immunity. All profiles and therapeutic implications shown are illustrative and require empirical validation. This is a conceptual schematic and contains no original patient or animal data.

Pulmonary TB commonly involves multiple lesions that differ in architecture, bacterial burden, inflammatory activity, cavitation status, vascular access, and antimicrobial exposure (2, 6, 9). Consequently, no single blood measurement can uniquely identify the biology of every lesion in an individual patient. Blood-based immunometabolic/death-mode signals may reflect overall disease activity or combined effects across lesions; they should be treated as exploratory correlates requiring prospective calibration against imaging and, when feasible, tissue-resolved data rather than lesion-level estimators (**Figure 5**) (14–16).

### 5.1 Lesion-resolved imaging and tissue-anchored validation

Longitudinal imaging is currently the most feasible way to monitor lesion heterogeneity in living patients. CT and PET/CT can assess lesion number, size, cavity formation, structural change, and metabolic activity over time (7, 59, 60). These modalities do not independently identify a specific cell-death mechanism; however, they provide the anatomical and temporal backbone for testing whether axis-associated signals map to lesion trajectories (**Figure 5**).

Where clinically indicated and ethically feasible, surgical specimens, biopsies, bronchoalveolar lavage samples, sputum-derived material, and archived tissue may support tissue-anchored validation. The assay must match the inference being made: spatial transcriptomics can localize pathway-relevant neighborhoods, while protein/biochemical/structural *in situ* assays are needed for pathway engagement or mechanism-specific execution readouts (e.g., lipid peroxidation, extracellular chromatin–granule–protein structures, or same-cell multi-pathway coordination) (10, 11). Spot-based spatial transcriptomics should be interpreted as neighborhood-level association, not same-cell co-expression.

A rigorous translational study should test whether candidate signals improve prediction/mechanistic inference for lesion progression, treatment response, functional recovery, or relapse beyond conventional clinical, microbiological, and radiological measures.

### 5.2 Blood biomarkers: candidate correlates, not lesion-level estimators

Blood biomarkers remain attractive due to accessibility and scalability (14, 15, 61, 62). Consistent with **Figure 5**, they should be treated as exploratory correlates until prospectively calibrated against predefined lesion-level imaging outcomes and, where available, tissue-resolved reference measurements.

Prospective studies should evaluate whether blood signals correlate with predefined lesion-resolved imaging endpoints (e.g., cavity volume change, lesion expansion, persistent PET activity, delayed radiological resolution), delayed microbiological response, and post-treatment relapse. A clinically useful biomarker should demonstrate analytical validity, reproducibility, and incremental predictive value beyond established variables (e.g., baseline cavitation, bacterial burden, drug resistance, HIV status, diabetes, antimicrobial exposure, and standard inflammatory markers).

Blood-based assignment of lesion-mechanistic profiles (F/P/N) should be avoided unless the marker has been externally validated to predict a lesion-anchored phenotype (16) (**Table 4**).

**Table 4:** Comparison of necro-mosaic with existing tuberculosis host-response and stratified-HDT frameworks.

Framework	Unit of stratification	Primary biological resolution	Lesion versus blood resolution	Current validation status	Longitudinal/treatment linkage	Potential clinical use	Specific contribution of Necro-Mosaic and remaining limitation
Blood transcriptional signatures (e.g., diagnostic, risk, or treatment-response signatures)	Individual patient	Peripheral-blood gene-expression pattern	Blood/systemic; does not resolve individual lesions	Several signatures have external validation for diagnostic, prognostic, or treatment-monitoring applications	Available for selected signatures and cohort outcomes	Risk stratification, diagnosis support, treatment monitoring	Necro-Mosaic does not replace blood signatures. It addresses lesion heterogeneity that blood measurements may average across; however, it is not currently measurable from blood alone.
Blood-based immune/inflammatory endotypes	Individual patient or cohort subgroup	Circulating immune cells, cytokines, metabolites, or transcriptional programs	Predominantly blood/systemic	Emerging; reproducibility and clinical boundaries vary across cohorts	Some associations with severity or treatment response; limited lesion anchoring	Candidate enrichment for biomarker studies or stratified-HDT research	Necro-Mosaic evaluates local lesion-associated processes rather than assigning patient-level endotypes. Its relationship to blood endotypes requires prospective lesion-anchored calibration.
Clinical/radiological TB phenotypes	Individual patient; sometimes lesion	Symptoms, microbiology, CT/PET-CT, cavitation, extent of disease	Primarily patient-level; imaging can provide lesion-resolving structural information	Established for clinical management; mechanistic specificity limited	Strong for serial imaging and microbiological outcomes	Prognosis, treatment monitoring, trial endpoint definition	Necro-Mosaic can be linked to lesion trajectories measured by imaging, but imaging alone cannot assign F-, P-, or N-axis-associated activity.
Prior precision-medicine or stratified-HDT proposals	Individual patient subgroup defined by clinical, microbiological, immune, or biomarker features	Systemic host response and clinical risk	Mostly blood/systemic; limited direct lesion measurement	Conceptual to early translational; no validated axis-directed HDT allocation	Variable; generally limited prospective biomarker-guided intervention evidence	Trial enrichment and hypothesis-driven HDT development	Necro-Mosaic adds explicit requirements for lesion-resolved target evidence, local drug exposure, bacterial-control safeguards, and mechanism-specific validation before treatment allocation.
Necro-Mosaic framework	Individual lesion or local tissue neighborhood; not a patient-level endotype	F-axis-associated lipid-peroxidation injury; P-axis-associated coordinated inflammatory cell-death activity; N-axis-associated neutrophil extracellular activity	Lesion-aware; requires tissue-anchored assays and/or lesion-resolving imaging for validation	Hypothesis-generating; not a validated endotype, score, diagnostic test, or treatment-selection algorithm	Required but presently limited; must be tested using longitudinal imaging, microbiology, tissue anchoring, and perturbation	Research prioritization, lesion-mechanism testing, biomarker calibration, and preclinical/early translational HDT development	Provides a non-exclusive framework for mixed and indeterminate lesion profiles and explicitly separates capacity, engagement, and mechanism-specific causal evidence. Limitations include invasive sampling constraints, lesion heterogeneity, cross-sectional human datasets, and absent prospective therapeutic validation.

“Endotype” should be reserved for a clinically reproducible biological subgroup with validated prognostic or treatment implications. Necro-Mosaic is not currently a patient-level endotype framework.

### 5.3 Host-directed therapy: clinical evidence, rationale, and safety requirements

HDT aims to improve outcomes when added to effective antimicrobials by limiting harmful inflammation, supporting tissue repair, improving lesion function, or modifying host pathways relevant to bacterial control. However, existing TB HDT trials do not validate Necro-Mosaic axes for lesion-level treatment assignment or blood-based allocation of lesion mechanisms; they illustrate how intervention class, timing, exposure, endpoint selection, and safety monitoring influence interpretation.

Representative trials show heterogeneity in benefit and reinforce the need for target-engagement and exposure-aware design. For example: everolimus + CC-11050 was reported as safe with preliminary functional recovery signals but without definitive microbiological efficacy or lesion-mechanism validation (63); metformin did not establish standard-of-care benefit on sputum culture conversion and motivates further exposure/context and subgroup evaluation (64); adjunctive rosuvastatin was safe but showed limited overall culture-conversion benefit and no overall PET/CT imaging gain, with possible subgroup/lesion-specific interpretation required (65, 66); vitamin D results were heterogeneous and did not support uniform effect (67, 68). Other important HDT clinical trials, such as those evaluating adjunctive prednisone (69) or combined therapies for drug-resistant TB (70), further illustrate this complexity.

These studies should not be interpreted as evidence for or against F-, P-, or N-axis targeting. Rather, they show that plausible host-directed interventions can be neutral, subgroup-dependent, functionally limited, or safety-constrained when target engagement, lesion exposure, and validated enrichment are not established.

Any intervention that modifies inflammatory signaling, oxidative stress, iron metabolism, neutrophil function, extracellular chromatin, proteases, or cell-death pathways must be evaluated for the risk of impairing protective antimycobacterial immunity. TNF neutralization illustrates this principle: TNF contributes to immunopathology but is also required for granuloma organization and host control; blockade can increase TB reactivation/dissemination risk (25, 71).

A complementary lesion-aware therapeutic hypothesis is to limit uncontrolled lytic death when such death promotes release, transfer, or subsequent expansion of viable bacilli. In experimental systems, M. tuberculosis–induced necrosis of infected neutrophils can promote bacterial growth after uptake by macrophages (37). This does not establish a general human lesion mechanism, nor imply that preventing cell death is always beneficial; nonetheless, it separates the question from suppressing inflammation alone and motivates direct measurement of viable bacterial release/transfer, intracellular vs extracellular burden, efferocytic handling, lesion dissemination, and host-cell survival alongside injury and immune-function endpoints. In this context, purinergic signaling shaped by ectonucleotidase activity can act as an upstream coordinator linking local ATP–P2X7 signaling to necrotic outputs; consistent with this concept, CD39 limits P2RX7-driven lung necrotic lesions in severe experimental tuberculosis by restricting necrotic death–linked pathological expansion (5).

Accordingly, axis-linked interventions remain preclinical/early translational hypotheses until lesion-resolved evidence is available (**Figure 5**):

F-axis: require lesion-relevant lipid-peroxidation injury evidence, target engagement, adequate local exposure, and preserved bacterial control.

P-axis: require evidence that targeted inflammatory cell-death activity contributes to pathology independently of protective host defense.

N-axis: require separate evaluation of neutrophil recruitment; structurally defined NET-associated extracellular material and NET-associated extracellular effector persistence; NET-linked protease and oxidative pathways, including MPO/NE (e.g., ELANE) and NOX2/CYBB-associated signaling; matrix injury; bacterial containment; and secondary-infection/dissemination risk, with explicit safeguards demonstrating that intervention does not impair protective antimycobacterial immunity.

Candidate interventions should progress through mechanistic validation, lesion-resolved PK/PD, safety-focused early clinical studies, and biomarker-anchored, adequately powered randomized trials (**Figure 5**).

### 5.4 Spatial pharmacology and lesion-specific exposure

Lesion heterogeneity affects both local host biology and local drug exposure. Agents may differ in concentration, penetration, retention, and activity across cellular, necrotic, fibrotic, vascularized, and cavity-associated regions (8, 9). A target is not therapeutically actionable unless the intervention reaches the relevant tissue environment at an effective concentration and produces evidence of target engagement.

Spatial pharmacology should be integrated early using approaches including matrix-assisted laser desorption/ionization mass spectrometry imaging, laser-capture microdissection coupled with drug quantification, PET-based tracer methods, microdialysis where appropriate, and lesion-level PK/PD modeling (8, 9). These methods can establish whether a candidate intervention reaches the lesion region, achieves mechanism-consistent exposure, produces local target engagement, alters lesion-associated readouts, and improves lesion trajectory without compromising bacterial control. Lack of therapeutic effect may reflect inadequate lesion penetration rather than failure of the biological hypothesis.

### 5.5 Clinical research priorities and feasible staged study designs

The central translational question is not whether patients can currently be assigned to F-, P-, or N-dominant categories. The priority is whether lesion-anchored evidence of axis-associated processes improves prediction of lesion progression, functional recovery, treatment response, or relapse beyond established clinical, microbiological, and radiological measures (**Figure 5**).

Because pulmonary-lesion biopsy is not routine and may entail bleeding, pneumothorax, sampling bias, and infection-control concerns, serial tissue acquisition should not be assumed feasible. Accordingly, tissue-based measurements should be pursued only when clinically indicated or from archived surgical material. The main platform is longitudinal imaging with sputum/blood/respiratory-fluid sampling.

Stage 1: Prospective lesion-aware observational cohorts

Prospective multicenter cohorts of adults with microbiologically confirmed pulmonary TB receiving standard therapy. Baseline assessments should include: chest CT (± PET/CT in a nested substudy), sputum culture and molecular bacterial-load measures, routine blood sampling, clinical severity/comorbidity/drug resistance/antimicrobial exposure, lung-function, and standardized symptom and quality-of-life measures. Repeat imaging/microbiology at end of intensive phase, at treatment completion, and during post-treatment follow-up. BAL/sputum-derived material or clinically indicated tissue may provide exploratory anchoring.

Prespecified lesion-level outcomes: changes in cavity/lesion volume, PET activity, radiological progression/resolution. Patient-level outcomes: time to culture negativity, culture conversion at predefined timepoints, treatment failure, relapse-free cure, lung-function recovery, quality of life, and serious adverse events. Analyses should test incremental predictive value beyond baseline cavitation/bacterial burden/HIV/diabetes/drug resistance/antimicrobial exposure and conventional inflammatory markers, reporting negative/discordant/indeterminate results rather than forcing axis categorization.

Stage 2: Tissue-anchored and pharmacology-anchored mechanistic validation

Candidate biomarkers from Stage 1 undergo tissue-anchored validation where clinically available to determine whether the signal reflects (a) pathway capacity, (b) pathway engagement, (c) mechanism-specific execution, (d) bacterial localization, (e) local tissue injury, (f) lesion progression, or (g) systemic inflammation without lesion specificity. For therapeutics, this stage includes lesion-resolved exposure and target-engagement assessment; enrichment should not proceed until reproducible lesion-anchored links are established in independent datasets.

Stage 3: Biomarker-enrichment trials

Biomarker-enrichment designs follow analytical validation and prospective calibration against lesion-resolving outcomes. Randomization remains against standard therapy plus placebo/standard care. Enrichment criteria must be predefined *a priori* and should not be described as F-/P-/N endotypes unless they satisfy reproducibility, lesion anchoring, and predictive validity requirements (**Figure 5**).

Stage 4: Adaptive platform trials after validated enrichment

Adaptive platforms may be considered when multiple credible HDT candidates exist and enrichment criteria are validated. Studies may use shared control arms and prospectively defined stopping rules for futility, unacceptable safety signals, inadequate lesion exposure, absent target engagement, worsening bacterial control, increased dissemination/secondary infection, and lack of improvement in prespecified lesion/patient outcomes. Adaptation should follow predefined safety/efficacy rules rather than *post hoc* subgrouping. Even with validated lesion-anchored biomarkers, deterministic allocation requires replicated clinical benefit.

#### 5.5.1 Minimum safety dataset for axis-relevant HDT studies

Across stages, minimum safety monitoring should include serial sputum culture/molecular bacterial-load where feasible; surveillance for dissemination, treatment failure, relapse, and secondary infection; CBC, hepatic and renal function; inflammatory markers; and serial CT or PET/CT where feasible. Mechanism-specific monitoring should additionally include (**Table 5**):

**Table 5:** Translational implications of necro-mosaic axes: evidence, intervention maturity, and safety.

Axis	What can currently be measured	Candidate intervention direction	Development status/prior TB evidence	Principal safety concern	Minimum safety-monitoring package	Translational conclusion
F axis (F-axis-associated lipid peroxidation)	Direct tissue evidence should include orthogonal lipid-peroxidation readouts together with cell-context information; blood ferroptosis signatures are not lesion-specific	Modulate lipid peroxidation, antioxidant defense, or iron handling (e.g., ferroptosis inhibitors, antioxidant, or iron-modulating strategies)	Mainly mechanistic and preclinical rationale in TB; no validated ferroptosis-targeted HDT strategy exists for routine TB care	Iron manipulation may alter M. tuberculosis iron availability; systemic iron chelation may cause toxicity or impair host immunity	Serial sputum culture and/or molecular bacterial load; dissemination surveillance where clinically indicated; serial CT/PET-CT where feasible; complete blood count; hepatic/renal function; ferritin, transferrin saturation, and serum iron where iron handling is modulated; lesion-resolved exposure/target-engagement measures where feasible	Suitable for mechanistic investigation, not treatment selection; any intervention requires lesion-resolved PK/PD and evidence that benefit exceeds impairment of host defense
P axis (coordinated inflammatory cell-death activity)	Same-cell or protein-level evidence of coordinated pathway activation is required; spot-level co-occurrence and inflammatory cytokines are insufficient	Carefully targeted modulation of upstream inflammatory or convergence nodes; combined pathway inhibition should not be presumed beneficial	Predominantly conceptual or preclinical; TB-specific clinical strategy should not be described as PANoptosis-directed without mechanism-specific evidence	Suppression of inflammatory pathways may compromise protective antimycobacterial immunity; TNF blockade can increase the risk of TB reactivation and dissemination	Serial microbiology; clinical surveillance for dissemination or opportunistic infection; complete blood count and hepatic function; inflammatory markers; lesion imaging; immune-function measures where feasible; target-engagement assessment before dose escalation	High risk; test only after showing the target contributes to lesion damage independently of protective bacterial control
N axis (neutrophil extracellular activity)	Direct NET-structure evidence requires extracellular DNA structurally co-localized with CitH3 and neutrophil proteins; neutrophil abundance or PADI4/ELANE/MPO expression alone is insufficient	Context-dependent modulation of NET formation and extracellular effector persistence, including protease activity (ELANE/NE) and NADPH oxidase-linked oxidative pathways (CYBB/NOX2), extracellular-chromatin persistence, and matrix injury; DNase-based approaches remain hypothesis-driven	Hypothesis-generating in TB. No validated NET-directed HDT strategy exists. General DNase/NET-clearance concepts are extrapolated largely from non-TB systems; recent human tissue association does not constitute intervention evidence	NET inhibition may reduce antimicrobial containment; DNase/protease inhibition may alter local inflammatory signaling, host defense, and repair; potential for releasing viable bacilli depending on context	Serial microbiology and surveillance for secondary infection/dissemination; complete blood count with neutrophil count; inflammatory markers; serial lesion imaging for cavitation or structural progression; exploratory measurement of NET-associated extracellular material and neutrophil protease activity where feasible	Not uniformly protective; utility depends on demonstrating that persistent NET-associated material contributes to lesion damage in a defined context
Cross-cutting	Serial microbiology, imaging, pharmacology, safety, and lesion-anchored biomarker assessment rather than fixed axis assignment	Context-specific adjunctive HDT development guided by mechanism, exposure, and safety	Representative primary clinical studies of everolimus, CC-11050, metformin, rosuvastatin, vitamin D, and corticosteroids demonstrate heterogeneous efficacy and safety signals across TB populations and endpoints (see **Supplementary Table S2**). None tested Necro-Mosaic-based stratification, lesion-level axis assignment, or axis-directed HDT allocation.	Immunopathology reduction may conflict with antimicrobial host defense; off-target toxicity and lesion under-exposure remain key risks	Serial microbiology; clinical surveillance for dissemination, treatment failure, secondary infection, and serious adverse events; complete blood count; hepatic and renal function; inflammatory markers; serial CT/PET-CT where feasible; and lesion-resolved exposure/target-engagement measures where feasible.	Current clinical trials provide development context, not validation of Necro-Mosaic-guided treatment selection.

Candidate intervention directions are not recommendations for clinical use. They are hypotheses requiring target validation, lesion-resolved pharmacology, prospective safety assessment, and clinically meaningful efficacy endpoints. Abbreviations: CitH3, citrullinated histone H3; CT, computed tomography; DNA, deoxyribonucleic acid; ELANE, neutrophil elastase; F/P/N, F-axis-associated lipid peroxidation/P-axis-associated coordinated inflammatory cell-death activity/N-axis-associated neutrophil extracellular activity; HDT, host-directed therapy; MPO, myeloperoxidase; NET, neutrophil extracellular trap; PADI4, peptidyl arginine deiminase 4; PET, positron emission tomography; PK/PD, pharmacokinetics/pharmacodynamics; TB, tuberculosis; TNF, tumor necrosis factor.

F-axis: iron indices, oxidative-stress markers, validated target-engagement measures, bacterial burden, and off-target tissue toxicity assessment.

P-axis: immune-function measures, infection surveillance, bacterial burden, inflammatory activity, and unintended suppression of protective immune responses.

N-axis: neutrophil counts, secondary-infection surveillance, matrix-injury measures, protease activity, bacterial control, and exploratory measures of structurally defined NET-associated extracellular material.

Where feasible, lesion-resolved drug exposure and local target engagement should be measured using validated pharmacological approaches.

Until these validation stages are complete, Necro-Mosaic should be used to prioritize experiments, define evidentiary thresholds, and interpret lesion heterogeneity—not to guide individual HDT selection. (**Figure 5**).

## 6 Discussion: controversies, evidence gaps, and falsifiable priorities

Necro-Mosaic is intended to organize uncertainty rather than resolve it prematurely. Available evidence supports substantial heterogeneity among TB lesions and supports biological relevance of cell death, neutrophil-associated activity, metabolic stress, extracellular-matrix remodeling, and heterogeneous local drug exposure (2, 3, 6, 9). However, major uncertainties remain:

- execution-level distribution of axis-associated processes across human lesions;
- whether these processes are causal upstream contributors to destructive pathology versus downstream correlates;
- whether they can be modulated without compromising bacterial control; and
- how to interpret mixed, discordant, and indeterminate lesion profiles within a non-exclusive framework.

### 6.1 From spatial association to causal inference

Spatial co-localization is not causation. Axis-associated signals may reflect consequences of established necrosis/oxidative injury or reflect mixed cellular neighborhoods rather than coordinated execution in the same cells; similarly, NET-associated extracellular material may be downstream of tissue injury and failed resolution rather than a primary driver of matrix destruction (10, 11, 23).

Accordingly, causal interpretation should require the same logic specified in **Boxes 1**, **2** and summarized in **Figure 4**: separate capacity/engagement from mechanism-specific execution, and test causality with lesion-relevant outcomes using temporally and spatially appropriate readouts.

For clarity, the causal requirement can be summarized as three non-redundant criteria:

- Temporal linkage: axis-associated activity should precede lesion deterioration or trajectory change; cross-sectional data cannot establish ordering.
- Execution-level specificity: pathway transcripts, lineage/capacity markers, or marker abundance alone are insufficient—mechanism-discriminating structural/biochemical/protein-level and functional evidence is required.
- Perturbational support tied to prespecified outcomes: selective modulation should change predefined lesion-relevant outcomes (injury, necrosis/cavitation, bacterial burden, treatment response, functional recovery/relapse), ideally alongside region-matched evidence of target engagement and local exposure.

Mixed/indeterminate profiles are therefore not treated as analytic failure; they are expected consequences of non-exclusive axes and should be interpreted with evidence-tier constraints rather than with forced categorical assignment.

### 6.2 Model-system and sampling limitations

No experimental system fully recapitulates human pulmonary TB, especially the diversity of lesion architectures, treatment histories, immune states, and advanced cavitary phenotypes observed clinically (6). Conventional mouse strains often do not develop the organized caseous necrosis or cavitation characteristic of advanced human disease; C3HeB/FeJ mice can develop necrotic lesions but differ in immune composition, lesion evolution, and pharmacological context (13). Higher-cost models (rabbits, guinea pigs, zebrafish, nonhuman primates) provide complementary strengths yet differ in throughput, accessibility, immune biology, and genetic/measurement tools (6).

Human spatial datasets also have systematic limitations. Surgical specimens may be enriched for drug-resistant, treatment-refractory, or advanced destructive disease, potentially overrepresenting permissive trajectories (1). Cross-sectional sampling can misclassify dynamic or evolving biology as a single “axis pattern,” particularly when lesion stage, cellular composition, and execution-level evidence are not explicitly assessed.

To improve cross-study interpretability, future reports should at minimum specify: host species/model; tissue source/anatomical site; lesion morphology and disease stage; treatment status/drug resistance; bacterial burden and measurement method; imaging and assay resolution; spatial scale of inference; whether evidence reflects capacity/engagement/execution; and the limits on temporal/causal interpretation.

### 6.3 What findings would change the conclusions?

Necro-Mosaic would be strengthened if one or more candidate axes repeatedly satisfied execution-level reproducibility, temporal linkage to lesion trajectories, and causal perturbation in relevant models and/or human lesion datasets. Supportive evidence would include:

- Reproducible detection of execution-level axis-associated features in relevant lesion contexts, with explicit documentation of mixed/indeterminate profiles.
- Mechanism-specific execution evidence (not only abundance or transcript signatures).
- Longitudinal association between axis activity and subsequent lesion progression/resolution using lesion-resolved imaging, microbiological/pathological measures, and/or functional readouts.
- Causal modification of lesion-relevant outcomes after selective perturbation with appropriate region-matched engagement/exposure considerations.
- Preserved or improved bacterial control, acknowledging that tissue-injury–contributing pathways may also participate in protective immunity.
- Replication across evidence systems with transparent reporting of differences in anatomy, stage, host species, and treatment context.
- Lesion-resolved pharmacological feasibility showing that interventions reach and engage targets in the relevant lesion region.

Conversely, weakening or disconfirmation would include failure to reproduce execution-level association with lesion outcomes, non-specific execution markers that do not map to mechanism, lack of lesion-trajectory change after selective perturbation, or effects restricted to marker changes without meaningful changes in injury, bacterial control, treatment response, or functional outcomes.

### 6.4 Priority experiments

Below we list priority experiments that minimize common interpretive ambiguities and directly target the evidence gaps above, without repeating the general evidence-tier logic already stated in **Boxes 1**, **2**.

F-axis: distinguishing lipid-peroxidation injury from ferroptotic execution

Priority studies should separate lipid-peroxidation injury (oxidative lipid stress) from ferroptosis as a mechanism-specific executed death program. In human tissue, markers such as 4-HNE, MDA, oxidized-lipid probes, and lesion-context readouts should generally be interpreted as evidence of oxidative injury or F-axis engagement rather than definitive ferroptotic execution.

In matched experimental systems, stronger ferroptosis claims should require an orthogonal evidence panel for: iron and oxidized-phospholipid dependence; antioxidant-system logic (e.g., GPX4/related pathways); exclusion of plausible alternative death mechanisms; selective pharmacological/genetic inhibition with rescue; and preserved or improved bacterial control where relevant. Studies should then test whether the intervention alters necrotic expansion, matrix injury, bacterial burden, and lesion trajectories while directly measuring local redox/iron/oxygenation context and drug exposure. A fixed “hypoxic ferroptosis niche” should not be assumed without direct measurements.

P-axis: testing coordinated inflammatory cell-death convergence

Priority studies for the P axis should determine whether activated apoptotic, necroptotic, and pyroptotic execution branches occur within the same cells rather than appearing as adjacent or transcriptionally mixed neighborhoods. Same-cell co-detection of execution-associated features (e.g., cleaved caspase-8, activated MLKL, GSDMD cleavage) can support coordinated multi-branch activity, but does not alone establish PANoptosis as a universal discrete complex.

A stronger P-axis claim should include: high-resolution same-cell execution evidence; temporal linkage to restrictive–permissive trajectory change; perturbation of a proposed convergence node or defined branch; loss-of-function/rescue; lesion-relevant outcomes including bacterial burden and tissue injury; and safeguards demonstrating that protective antimicrobial immunity is not impaired.

N-axis: separating neutrophil localization, NET structures, and NETotic death

Priority N-axis work should map, separately: neutrophil localization; NET-associated signals; structurally defined extracellular NET material; persistence of extracellular-effector activity; matrix injury and cavitation; bacterial burden; and NETotic execution. In human tissue, structurally defined NET evidence should include extracellular DNA structurally co-localized with citrullinated histone H3 and neutrophil proteins (e.g., MPO or neutrophil elastase) with appropriate compartmental and non-NET extracellular DNA controls; such evidence supports NET structure assignment, not NETotic cell death. NETotic claims further require lytic, temporal, and functional evidence.

Longitudinal studies should test whether structurally defined NET-associated material precedes, follows, or is unrelated to remodeling/cavitation. Experimental perturbation should evaluate bacterial dissemination/secondary infection and host-cell survival together with matrix injury and lesion trajectory. DNase-mediated extracellular-DNA degradation and efferocytic removal should be treated as distinct biological modifiers rather than merged into a single “clearance capacity” concept.

#### 6.4.1 Spatial epigenetic regulation: explicitly hypothesis-generating

Spatial epigenetic regulation remains hypothesis-generating. Spatial ATAC-seq, spatial CUT&Tag/CUT&RUN, and multiplex protein approaches could test whether chromatin states precede and condition susceptibility to F-, P-, or N-axis execution in defined lesion contexts. Such work should include robust cell-type assignment and address alternative explanations such as lesion stage, treatment exposure, generalized necrosis/cell abundance, and fixation-related artifacts. Current broader evidence that inflammatory/metabolic stress shapes epigenetic states does not, by itself, demonstrate lesion-specific chromatin causally licenses PCD-associated execution programs in TB granulomas.

## 7 Conclusion

TB lesions are spatially heterogeneous ecosystems where immune activation, tissue injury, bacterial persistence, tissue remodeling, and drug exposure can diverge across lesions and over time. Necro-Mosaic provides a non-exclusive and falsifiable framework for investigating ferroptosis-associated, PANoptosis-like, and NET-associated processes as candidate contributors to this heterogeneity.

The framework does not define clinical endotypes, lesion scores, biomarkers, or treatment-selection rules. Its utility will depend on whether axis-associated processes can be measured at execution level, linked prospectively to lesion trajectories, interpreted appropriately in mixed/indeterminate profiles, and modulated safely without compromising bacterial control. Until such evidence is available, the highest priorities remain lesion-resolved imaging, tissue-anchored molecular analysis, spatial pharmacology, and carefully designed longitudinal/perturbational studies (8, 9, 16, 24).

## References

[1] Cohen SB Gern BH Urdahl KB . The tuberculous granuloma and preexisting immunity. Annu Rev Immunol. (2022) 40:589–614. doi: 10.1146/annurev-immunol-093019-125148 35130029

[2] Lin PL Ford CB Coleman MT Myers AJ Gawande R Ioerger T . Sterilizing immunity against Mycobacterium tuberculosis infection follows a local, not systemic, pattern. PloS Pathog. (2014) 10:e1003873. doi: 10.1371/journal.ppat.100387324465206

[3] McCaffrey EF Donato M Keren L Chen Z Delmastro A Fitzpatrick MB . The immunoregulatory landscape of human tuberculosis granulomas. Nat Immunol. (2022) 23:318–29. doi: 10.1038/s41590-021-01121-x 35058616 PMC8810384

[4] Tateosian NL Morelli MP Pellegrini JM García VE . Beyond the clinic: The activation of diverse cellular and humoral factors shapes the immunological status of patients with active tuberculosis. Int J Mol Sci. (2023) 24:5033. doi: 10.3390/ijms24055033 36902461 PMC10002939

[5] Almeida-Santos G Santiago-Carvalho I Almeida FM Bomfim CCB Santos e Silva JC Cantarini DG . CD39 regulates P2RX7-mediated lung necrotic lesions in severe experimental tuberculosis. Mucosal Immunol. (2026) 19:100333. doi: 10.1016/j.mucimm.2026.03.007 41839294 PMC13148415

[6] Hunter L Ruedas-Torres I Agulló-Ros I Rayner E Salguero FJ . Comparative pathology of experimental pulmonary tuberculosis in animal models. Front Vet Sci. (2023) 10:1264833. doi: 10.3389/fvets.2023.1264833 37901102 PMC10602689

[7] Medrano JM Maiello P Rutledge T Tomko J Rodgers MA Fillmore D . Characterizing the spectrum of latent Mycobacterium tuberculosis in the cynomolgus macaque model: Clinical, immunologic, and imaging features of evolution. J Infect Dis. (2023) 227:592–601. doi: 10.1093/infdis/jiac504 36611221 PMC9927077

[8] Cicchese JM Dartois V Kirschner DE Linderman JJ . Both pharmacokinetic variability and granuloma heterogeneity impact the ability of the first-line antibiotics to sterilize tuberculosis granulomas. Front Pharmacol. (2020) 11:333. doi: 10.3389/fphar.2020.00333 32265707 PMC7105635

[9] Prideaux B Via LE Zimmerman MD Eum S Sarathy J O'Brien P . The association between sterilizing activity and drug distribution into tuberculosis lesions. Nat Med. (2015) 21:1223–7. doi: 10.1038/nm.3937 26343800 PMC4598290

[10] Qiu X Zhong P Yue L Li C Yun Z Si G . Spatial transcriptomic sequencing reveals immune microenvironment features of Mycobacterium tuberculosis granulomas in lung and omentum. Theranostics. (2024) 14:6185–201. doi: 10.7150/thno.99038 39431015 PMC11488093

[11] Sawyer AJ Patrick E Edwards J Wilmott JS Fielder T Yang Q . Spatial mapping reveals granuloma diversity and histopathological superstructure in human tuberculosis. J Exp Med. (2023) 220:e20221392. doi: 10.1084/jem.20221392 36920308 PMC10035589

[12] Wang L Ma H Wen Z Niu L Chen X Liu H . Single-cell RNA-sequencing reveals heterogeneity and intercellular crosstalk in human tuberculosis lung. J Infection. (2023) 87:373–84. doi: 10.1016/j.jinf.2023.09.004 37690670

[13] Seto S Omori S Nakamura H Hijikata M Keicho N . Single-cell transcriptomic profiling reveals a novel signature of necrotizing granulomatous lesions in the lungs of Mycobacterium tuberculosis-infected C3HeB/FeJ mice. Front Immunol. (2025) 16:1624072. doi: 10.3389/fimmu.2025.1624072 40843005 PMC12364640

[14] Sweeney TE Braviak L Tato CM Khatri P . A 3-gene inflammatory signature for tuberculosis diagnosis and treatment monitoring. Lancet Respir Med. (2016) 4:213–24. doi: 10.1016/S2213-2600(16)00048-5 PMC4838193 26907218

[15] Penn-Nicholson A Mbandi SK Thompson E Mendelsohn SC Suliman S Chegou NN . RISK6, a 6-gene transcriptomic signature of TB disease risk, diagnosis and treatment response. Sci Rep. (2020) 10:8629. doi: 10.1038/s41598-020-65043-8 32451443 PMC7248089

[16] DiNardo AR Nishiguchi T Grimm SL Schlesinger LS Graviss EA Cirillo JD . Tuberculosis endotypes to guide stratified host-directed therapy. Med. (2021) 2:217–32. doi: 10.1016/j.medj.2020.11.003 34693385 PMC8530349

[17] Amaral EP Namasivayam S Queiroz ATL Fukutani E Hilligan KL Aberman K . BACH1 promotes tissue necrosis and Mycobacterium tuberculosis susceptibility. Nat Microbiol. (2024) 9:120–35. doi: 10.1038/s41564-023-01523-7 38066332 PMC10769877

[18] Roy A Agnivesh PK Sau S Kumar S Kalia NP . Tweaking host immune responses for novel therapeutic approaches against Mycobacterium tuberculosis. Drug Discov Today. (2023) 28:103693. doi: 10.1016/j.drudis.2023.103693 37390961

[19] Christgen S Zheng M Kesavardhana S Karki R Kanneganti TD . Identification of the PANoptosome: A molecular platform triggering pyroptosis, apoptosis, and necroptosis (PANoptosis). Front Cell Infect Microbiol. (2020) 10:237. doi: 10.3389/fcimb.2020.00237 32547960 PMC7274033

[20] Karki R Sharma BR Tuladhar S Williams EP Zalduondo L Samir P . PANoptosis: Cell death, inflammatory pathologies, and therapeutics. Trends Immunol. (2021) 42:673–85. doi: 10.1016/j.it.2021.06.002 34253468

[21] Fisher KL Mpotje T Nargan K Moodley D Rajkumar-Bhugeloo K Baiyegunhi OO . NETosis associates with human TB lung tissue destruction and disease pathogenesis. EMBO Mol Med. (2026) 18:2547–72. doi: 10.1038/s44321-026-00435-3 42230989 PMC13365388

[22] Farrera C Fadeel B . Macrophage clearance of neutrophil extracellular traps is a silent process. J Immunol. (2013) 191:2647–56. doi: 10.4049/jimmunol.1300436 23904163

[23] Pyle CJ Wang L Beerman RW Jain V Ohman HKE Thompson BA . Paired single-cell and spatial transcriptional profiling reveals a central osteopontin macrophage response mediating tuberculous granuloma formation. mBio. (2025) 16:e01559-25. doi: 10.1128/mbio.01559-25 40772762 PMC12421895

[24] Lange C Aarnoutse R Chesov D van Crevel R Gillespie SH Grobbel HP . Perspective for precision medicine for tuberculosis. Front Immunol. (2020) 11:566608. doi: 10.3389/fimmu.2020.566608 33117351 PMC7578248

[25] Keane J Gershon S Wise RP Mirabile-Levens E Kasznica J Schwieterman WD . Tuberculosis associated with infliximab, a tumor necrosis factor alpha-neutralizing agent. N Engl J Med. (2001) 345:1098–104. doi: 10.1056/NEJMoa011110 11596589

[26] Lyu J Narum DE Baldwin SL Larsen SE Coler RN . Understanding the development of tuberculous granulomas: Insights into host protection and pathogenesis, a review in humans and animals. Front Immunol. (2024) 15:1427559. doi: 10.3389/fimmu.2024.1427559 39717773 PMC11663721

[27] Park JH Shim D Kim KES Lee W Shin SJ . Understanding metabolic regulation between host and pathogens: New opportunities for the development of improved therapeutic strategies against Mycobacterium tuberculosis infection. Front Cell Infect Microbiol. (2021) 11:635335. doi: 10.3389/fcimb.2021.635335 33796480 PMC8007978

[28] McCaffrey EF Delmastro AC Fitzhugh I Ranek JS Douglas S Peters JM . The immunometabolic topography of cellular organization and bacterial control in tuberculosis granulomas. Nat Immunol (2026) 27:867–80. doi: 10.1038/s41590-026-02431-8 PMC13361869 41731147

[29] Cronan MR . In the thick of it: Formation of the tuberculous granuloma and its effects on host and therapeutic responses. Front Immunol. (2022) 13:820134. doi: 10.3389/fimmu.2022.820134 35320930 PMC8934850

[30] Cronan MR Hughes EJ Brewer WJ Viswanathan G Hunt EG Singh B . A non-canonical type 2 immune response coordinates tuberculous granuloma formation and epithelialization. Cell. (2021) 184:1757–1774.e14. doi: 10.1016/j.cell.2021.02.046 33761328 PMC8055144

[31] Camarasa TMN Iseppi L Schreiner D King CG . Tertiary lymphoid structures in tuberculosis: Persistence, protection, and pathology. Immunol Rev. (2025) 333:e70055. doi: 10.1111/imr.70055 40815083 PMC12356069

[32] Pisu D Sunny PS Nelson ML Junecko BF Venugopalan R Rodgers MA . Spatially-distinct programming of macrophage diversity within the granulomas of Mycobacterium tuberculosis infected nonhuman primates. In: Biorxiv Cold Spring Harbor, NY, USA: Cold SpringHarbor Laboratory. (2025). doi: 10.1101/2025.06.12.659348

[33] Lefrançais E Hudrisier D Neyrolles O Behar SM Ernst JD . Finding and filling the knowledge gaps in mechanisms of T cell-mediated TB immunity to inform vaccine design. Nat Rev Immunol. (2025) 25:798–815. doi: 10.1038/s41577-025-01192-z 40514544 PMC12720485

[34] Niewold P IJsselsteijn ME Dijkman K de Miranda N Ottenhoff THM Verreck FAW . NK cell-macrophage interactions in granulomas correlate with limited tuberculosis pathology. PloS Pathog. (2025) 21:e1012980. doi: 10.1371/journal.ppat.1012980 40749077 PMC12360647

[35] Genoula M Marín Franco JL Maio M Dolotowicz B Ferreyra M Milillo MA . Fatty acid oxidation of alternatively activated macrophages prevents foam cell formation, but Mycobacterium tuberculosis counteracts this process via HIF-1α activation. PloS Pathog. (2020) 16:e1008929. doi: 10.1371/journal.ppat.1008929 33002063 PMC7553279

[36] Luo G Wen Y Wang M Wang H Li D Liao M . A granulin-positive macrophage subtype in mycobacterial granulomas alleviates tissue damage by limiting excessive inflammation. Proc Natl Acad Sci. (2025) 122:e2413946122. doi: 10.1073/pnas.2413946122 40729382 PMC12337285

[37] Dallenga T Repnik U Corleis B Eich J Reimer R Griffiths GW . M. tuberculosis-induced necrosis of infected neutrophils promotes bacterial growth following phagocytosis by macrophages. Cell Host Microbe. (2017) 22:519–30. doi: 10.1016/j.chom.2017.09.003 29024644

[38] Ong CW Elkington PT Brilha S Ugarte-Gil C Tome-Esteban MT Tezera LB . Neutrophil-derived MMP-8 drives AMPK-dependent matrix destruction in human pulmonary tuberculosis. PloS Pathog. (2014) 11(5):e1004917. doi: 10.1371/journal.ppat.1004917 25996154 PMC4440706

[39] Shang X Maimaiti N Fan J Wang L Wang Y Sun H . Triggering receptor expressed on myeloid cells 2 mediates the involvement of M2-type macrophages in pulmonary tuberculosis infection. J Inflammation Res. (2024) 17:1919–28. doi: 10.2147/JIR.S435216 38562656 PMC10982454

[40] Mitsui Y Satoh T . Functional diversity of disorder-specific macrophages involved in various diseases. Inflammation Regeneration. (2025) 45:29. doi: 10.1186/s41232-025-00390-5 41035106 PMC12487080

[41] Nakamizo S Kabashima K . Metabolic reprogramming and macrophage polarization in granuloma formation. Int Immunol. (2024) 36:329–38. doi: 10.1093/intimm/dxae013 38441292

[42] Pan J Chang Z Zhang X Dong Q Zhao H Shi J . Research progress of single-cell sequencing in tuberculosis. Front Immunol. (2023) 14:1276194. doi: 10.3389/fimmu.2023.1276194 37901241 PMC10611525

[43] DiNardo AR Rajapakshe K Nishiguchi T Grimm SL Mtetwa G Dlamini Q . DNA hypermethylation during tuberculosis dampens host immune responsiveness. J Clin Invest. (2020) 130:3113–23. doi: 10.1172/JCI134622 32125282 PMC7260034

[44] Elkington PT D'Armiento JM Friedland JS . Tuberculosis immunopathology: The neglected role of extracellular matrix destruction. Sci Transl Med. (2011) 3:71ps6. doi: 10.1126/scitranslmed.3001847 21346167 PMC3717269

[45] Kubo T Hirohashi Y Tsukahara T Kanaseki T Murata K Hasegawa T . Epithelioid granulomatous lesions express abundant programmed death ligand-1 (PD-L1): A discussion of adverse events in anti-PD-1 antibody-based cancer immunotherapy. Hum Vaccines Immunotherapeutics. (2021) 17:1940–2. doi: 10.1080/21645515.2020.1870364 33570446 PMC8189068

[46] Le Rochais M Hémon P Ben-guigui D Garaud S Le Dantec C Pers JO . Deciphering the maturation of tertiary lymphoid structures in cancer and inflammatory diseases of the digestive tract using imaging mass cytometry. Front Immunol. (2023) 14:1147480. doi: 10.3389/fimmu.2023.1147480 37143660 PMC10151544

[47] Klepp LI Blanco FC Bigi MM Vázquez CL García EA Sabio García J . B cell and antibody responses in bovine tuberculosis. Antibodies. (2024) 13:84. doi: 10.3390/antib13040084 39449326 PMC11503302

[48] Su GL Zhang MJ Li H Sun ZJ . Dissecting tertiary lymphoid structures in cancer: Maturation, localization and density. Theranostics. (2025) 15:9459–85. doi: 10.7150/thno.113940 41041059 PMC12486156

[49] Zhang Y Li L Sheng ZK Rao YF Zhu X Pang Y . Increased tertiary lymphoid structures are associated with exaggerated lung tissue damage in smokers with pulmonary tuberculosis. BioMed Environ Sci. (2025) 38:810–8. doi: 10.3967/bes2024.168 40820247

[50] Devi A Pahuja I Singh SP Verma A Bhattacharya D Bhaskar A . Revisiting the role of mesenchymal stem cells in tuberculosis and other infectious diseases. Cell Mol Immunol. (2023) 20:600–12. doi: 10.1038/s41423-023-01028-7 37173422 PMC10176304

[51] Yang Q Zhou Y Farooq W Liu Q Duan J Xing L . The immunomodulatory effects of mesenchymal stem cells on THP-1-derived macrophages against Mycobacterium tuberculosis H37Ra infection. Tuberculosis. (2025) 150:102593. doi: 10.1016/j.tube.2024.102593 39709721

[52] Song L Zhang D Wang H Xia X Huang W Gonzales J . Automated quantitative assay of fibrosis characteristics in tuberculosis granulomas. Front Microbiol. (2023) 14:1301141. doi: 10.3389/fmicb.2023.1301141 38235425 PMC10792068

[53] Cross A Shelley J Newman R Strid J Denton AE . Role of fibroblast-immune crosstalk in kidney, lung, and skin tertiary lymphoid structures. Immunol Rev. (2025) 334:e70059. doi: 10.1111/imr.70059 40884054 PMC12397720

[54] Lu Z Zhang Y Zhong Y Qiang L Ge P Lei Z . A bacterial effector manipulates host lysosomal protease activity-dependent plasticity in cell death modalities to facilitate infection. Proc Natl Acad Sci. (2025) 122:e2406715122. doi: 10.1073/pnas.2406715122 39964716 PMC11874418

[55] Fonseca KL Lozano JJ Despuig A Habgood-Coote D Sidorova J Aznar D . Unravelling the transcriptome of the human tuberculosis lesion and its clinical implications. Nat Commun. (2025) 16:5028. doi: 10.1038/s41467-025-60255-w 40447570 PMC12125219

[56] Batista LAF Silva KJ Silva LM de Moura YF Zucchi FC . Tuberculosis: A granulomatous disease mediated by epigenetic factors. Tuberculosis. (2020) 123:101943. doi: 10.1016/j.tube.2020.101943 32741528

[57] Khadela A Chavda VP Postwala H Shah Y Mistry P Apostolopoulos V . Epigenetics in tuberculosis: Immunomodulation of host immune response. Vaccines. (2022) 10:1740. doi: 10.3390/vaccines10101740 36298605 PMC9611989

[58] Pellegrini JM Tateosian NL Morelli MP Garcia VE . Shedding light on autophagy during human tuberculosis. A long way to go. Front Cell Infect Microbiol. (2021) 11:820095. doi: 10.3389/fcimb.2021.820095 35071056 PMC8769280

[59] Chen RY Dodd LE Lee M Paripati P Hammoud DA Mountz JM . PET/CT imaging correlates with treatment outcome in patients with multidrug-resistant tuberculosis. Sci Transl Med. (2014) 6:265ra166. doi: 10.1126/scitranslmed.3009501 25473034 PMC5567784

[60] Malherbe ST Shenai S Ronacher K Loxton AG Dolganov G Kriel M . Persisting positron emission tomography lesion activity and Mycobacterium tuberculosis mRNA after tuberculosis cure. Nat Med. (2016) 22:1094–100. doi: 10.1038/nm.4177 27595324 PMC5053881

[61] Schiff HF Walker NF Ugarte-Gil C Tebruegge M Manousopoulou A Garbis SD . Integrated plasma proteomics identifies tuberculosis-specific diagnostic biomarkers. JCI Insight. (2024) 9:e173273. doi: 10.1172/jci.insight.173273 38512356 PMC11141874

[62] Yu Y Jiang XX Li JC . Biomarker discovery for tuberculosis using metabolomics. Front Mol Biosci. (2023) 10:1099654. doi: 10.3389/fmolb.2023.1099654 36891238 PMC9986447

[63] Wallis RS Ginindza S Beattie T Arjun N Likoti M Edward VA . Adjunctive host-directed therapies for pulmonary tuberculosis: A prospective, open-label, phase 2, randomised controlled trial. Lancet Respir Med. (2021) 9:897–908. doi: 10.1016/S2213-2600(20)30448-3 33740465 PMC8332197

[64] Padmapriydarsini C Mamulwar M Mohan A Shanmugam P Gomathy NS Mane A . Randomized trial of metformin with anti-tuberculosis drugs for early sputum conversion in adults with pulmonary tuberculosis. Clin Infect Dis. (2022) 75:425–34. doi: 10.1093/cid/ciab964 34849651 PMC9427151

[65] Cross GB Sari IP Kityo C Lu Q Pokharkar Y Moorakonda RB . Rosuvastatin adjunctive therapy for rifampicin-susceptible pulmonary tuberculosis: A phase 2b, randomised, open-label, multicentre trial. Lancet Infect Dis. (2023) 23:911–21. doi: 10.1016/S1473-3099(23)00067-1 36966799

[66] Cross GB Sari IP Burkill SM Yap CW Nguyen H Quyet D . PET-CT outcomes from a randomised controlled trial of rosuvastatin as an adjunct to standard tuberculosis treatment. Nat Commun. (2024) 15:10475. doi: 10.1038/s41467-024-54419-3 39622823 PMC11611914

[67] Martineau AR Timms PM Bothamley GH Hanifa Y Islam K Claxton AP . High-dose vitamin D3 during intensive-phase antimicrobial treatment of pulmonary tuberculosis: A double-blind randomised controlled trial. Lancet. (2011) 377:242–50. doi: 10.1016/S0140-6736(10)61889-2 21215445 PMC4176755

[68] Jolliffe DA Ganmaa D Wejse C Raqib R Haq MA Salahuddin N . Adjunctive vitamin D in tuberculosis treatment: Meta-analysis of individual participant data. Eur Respir J. (2019) 53:1802003. doi: 10.1183/13993003.02003-2018 30728208

[69] Meintjes G Stek C Blumenthal L Thienemann F Schutz C Buyze J . Prednisone for the prevention of paradoxical tuberculosis-associated IRIS. N Engl J Med. (2018) 379:1915–25. doi: 10.1056/NEJMoa1800762 30428290

[70] Tukvadze N Mashatole S Jacobs A Ngwanto T Heinrich N Van Rie A . DRTB-HDT: A randomized controlled trial of two adjunctive host-directed therapies in rifampin-resistant tuberculosis. BMC Infect Dis. (2025) 25:768. doi: 10.1186/s12879-025-11072-5 40437360 PMC12117953

[71] Flynn JL Gideon HP Mattila JT Lin PL . Immunology studies in non-human primate models of tuberculosis. Immunol Rev. (2015) 264:60–73. doi: 10.1111/imr.12258 25703552 PMC4339213

