# Supplementary material for: Necro-mosaic: a lesion-aware framework for testing programmed cell-death-associated mechanisms in tuberculosis
Source: Front Immunol. 2026 Oct 5;17:1933352. doi: 10.3389/fimmu.2026.1933352 (PMC13648034; doi:10.3389/fimmu.2026.1933352)
Supplement: Supplementary file 1 [file Table1.docx]

Supplementary Table S1. Minimum axis-specific validation panel and predefined falsification rules

| **Axis** | **Positive and negative controls** | **Minimum spatial/structural resolution** | **Orthogonal readouts** | **Temporal design** | **Perturbation/rescue requirement** | **Predefined falsification rule** |
| --- | --- | --- | --- | --- | --- | --- |
| F-axis-associated lipid-peroxidation injury | Positive: validated ferroptosis-compatible experimental control. Negative: antioxidant/iron-independent oxidative injury and alternative cell-death controls | Cell- and lesion-region-resolved; report relation to necrosis, bacteria, iron/redox state, and drug exposure | Lipid-peroxidation assay plus oxidized phospholipids, iron dependence, GPX4 or related antioxidant-system measures, and alternative-pathway assessment | Demonstrate whether F-axis-associated injury precedes, accompanies, or follows necrotic expansion | Pharmacological and/or genetic inhibition with target engagement; rescue required for a mechanism-specific ferroptosis claim | If intervention reduces lipid-oxidation markers without reducing tissue injury or altering lesion trajectory, F-axis activity is not supported as a causal driver. If benefit worsens bacterial control, the mechanism is context-dependent rather than uniformly pathogenic. |
| P-axis-associated coordinated inflammatory cell-death activity | Positive: model with experimentally induced coordinated apoptotic, necroptotic, and pyroptotic activity. Negative: isolated single-branch activation and mixed-cell-neighborhood control | Same-cell protein-level resolution; spot-level transcriptomic co-occurrence is insufficient | Activated/cleaved CASP8, MLKL, GSDMD and relevant inflammatory outputs; common-node assessment; functional cell-death readouts | Determine temporal ordering of multi-branch activity relative to tissue injury and bacterial change | Perturb proposed common regulatory node; include loss-of-function and rescue; verify effects are not limited to one branch | If multi-branch markers persist independently, or common-node perturbation does not alter coordinated activity and lesion outcome, an integrated PANoptotic mechanism is not supported. |
| N-axis-associated neutrophil extracellular activity | Positive: validated NET-structure control. Negative: extracellular DNA from generalized necrosis/apoptosis, isotype/antibody controls, and non-neutrophil DNA controls | Tissue-compartment-resolved imaging of extracellular DNA, CitH3, and neutrophil proteins; distinguish cavity-associated, airway-facing, necrosis-adjacent, and ECM-remodeling regions empirically | Structural co-localization of extracellular DNA, CitH3, and neutrophil proteins; matrix-protease activity; bacterial localization; separate DNase and efferocytosis measures | Test whether structurally defined NET-associated material precedes, follows, or is unrelated to matrix injury/cavitation | Selectively modulate a defined N-axis component (e.g., MPO/ELANE/CYBB-associated neutrophil effector pathways and/or NET-associated extracellular effector persistence); measure bacterial burden, dissemination, secondary infection, matrix injury, and lesion trajectory | If modulation reduces NET-associated signals/structures without changing tissue outcomes, N-axis activity is not supported as a lesion driver. If tissue injury improves but bacterial control worsens, a uniformly harmful interpretation is falsified. |
| Spatial epigenetic hypothesis | Positive: technically validated chromatin-accessibility/histone-mark control. Negative: cell-composition and fixation/processing controls | Cell-type and lesion-region-resolved chromatin mapping | Spatial ATAC-seq and/or CUT&Tag/CUT&RUN integrated with multiplex protein and cell-state data | Establish whether chromatin-state change precedes axis engagement/execution | Perturb candidate chromatin regulator and assess downstream axis-related readouts and lesion outcome | If chromatin states do not precede or modify subsequent axis engagement after adjustment for cell composition and lesion stage, epigenetic priming should not be retained as a causal explanation. |

Note. A marker should not be elevated from capacity or engagement to mechanism-specific execution without the indicated controls and orthogonal validation. “NET-associated extracellular structures” and “NETotic cell death” are distinct claims.

Supplementary Table S2. Representative clinical studies of host-directed therapies in tuberculosis and implications for lesion-aware development.

| **Intervention** | **Representative primary study/design** | **Population and principal endpoint** | **Main efficacy signal** | **Safety signal/limitation** | **Relevance to Necro-Mosaic** |
| --- | --- | --- | --- | --- | --- |
| Everolimus | Wallis et al., 2021; prospective, open-label, phase 2 randomized trial | Adults with pulmonary TB; safety and preliminary efficacy | Preliminary functional recovery signal, including FEV1 recovery; definitive microbiological benefit not established | Generally safe and reasonably tolerated in the trial; immunomodulation requires infection surveillance | Did not measure or stratify F/P/N axes; supports functional endpoints in future lesion-aware studies |
| CC-11050 | Wallis et al., 2021; prospective, open-label, phase 2 randomized trial | Adults with pulmonary TB; safety and preliminary efficacy | Preliminary functional recovery signal, including FEV1 recovery; no definitive axis-specific or microbiological conclusion | Generally safe and reasonably tolerated; inflammatory modulation may affect host defence | Did not test P-axis activity or lesion-resolved target engagement |
| Metformin | Padmapriydarsini et al., 2022; randomized pulmonary-TB trial | Adults with pulmonary TB; two-month culture conversion | Did not establish metformin as standard adjunctive therapy or accelerate culture conversion overall; supports further study of inflammatory, imaging, and host-modifier effects | Glycaemic status, renal function, and drug exposure require monitoring | Does not validate F-axis modulation; candidate contextual metabolic intervention only |
| Rosuvastatin | Cross et al., 2023; phase 2b randomized, open-label, multicentre trial | Rifampicin-susceptible pulmonary TB; culture conversion | Safe but no substantive culture-conversion benefit in the overall population | Monitor hepatic enzymes, creatine kinase, and drug interactions; PET-CT analysis showed no overall imaging benefit | Demonstrates that plausible HDT may be neutral without validated enrichment or target engagement |
| Vitamin D3 | Martineau et al., 2011; multicentre randomized placebo-controlled trial | Smear-positive pulmonary TB; time to culture conversion | No overall acceleration of culture conversion; possible genotype-defined subgroup signal | Monitor calcium and vitamin-D status; benefit not uniform | Does not support generic immune-endotype allocation; subgroup findings require prospective confirmation |
| Corticosteroid/prednisone | Meintjes et al., 2018; randomized placebo-controlled trial | HIV-associated TB; prevention of paradoxical TB-IRIS | Reduced TB-IRIS incidence in the defined HIV/ART clinical context | Immunosuppression and infection risks require careful indication-specific use | Demonstrates that HDT benefit may be context-specific; not evidence for pulmonary-lesion F/P/N allocation |
| Protocol-defined HDT trial: DRTB-HDT | Tukvadze et al., 2025; randomized controlled trial protocol | Rifampin-resistant TB; protocol-defined safety, microbiological, and host-directed endpoints | Protocol paper only; no efficacy conclusion should be drawn before trial completion | Requires prespecified microbiological, safety, pharmacology, and stopping-rule monitoring | Useful for prospective incorporation of lesion imaging and exploratory biomarker calibration, but not for treatment allocation or framework validation |

Note. These studies did not test Necro-Mosaic axis assignment, lesion-level endotyping, or axis-directed treatment selection. Trial findings should therefore be interpreted as clinical context for staged HDT development rather than validation of the proposed framework.

Supplementary Table S3. Causal-validation architecture for testing Necro-Mosaic hypotheses in tuberculosis lesions

| **Causal level** | **Core question** | **Minimum evidence and measurement approach** | **Key competing explanations/confounders** | **Required design feature** | **Interpretation if criterion is not met** |
| --- | --- | --- | --- | --- | --- |
| Lesion context | Are local conditions associated with the candidate axis? | Measure oxygenation, nutrient/lipid context, redox/iron state, ECM remodeling, vascular access, bacterial location/burden, and local drug exposure using lesion-resolved imaging, spatial assays, pathology, or pharmacology | Disease stage; treatment exposure; lesion morphology; species/model differences; tissue-processing artifacts | Report tissue source, host species, treatment status, lesion stage, anatomical context, and spatial resolution | Contextual association only; cannot establish axis activation or causal direction |
| Cellular capacity | Are relevant cells capable of engaging the candidate process? | Cell identity plus susceptibility-related transcripts/proteins: e.g., ACSL4/GPX4-related biology for F axis; pathway components for P axis; neutrophil/PADI4/ELANE/MPO markers for N axis | Cell-abundance effects; altered cell composition; dissociation bias; spot contamination; generalized inflammatory activation | Single-cell or spatial cell-type assignment with appropriate negative cellular comparators | Capacity only; does not establish pathway engagement or execution |
| Pathway engagement | Is there biochemical or protein-level activity consistent with the axis? | Orthogonal protein, biochemical, and spatial readouts; same-cell resolution where required | Non-specific oxidative injury; independent pathway activity in neighboring cells; extracellular DNA from non-NET injury | Multiplex assays, structural context, assay-positive and assay-negative controls | Engagement or association only; cannot establish a specific death mechanism |
| Mechanism-specific execution | Is the proposed process specifically executed? | F axis: iron/oxidized-phospholipid dependence, antioxidant-system involvement, alternative-pathway exclusion, and rescue in matched experimental systems. P axis: same-cell coordinated multi-branch activity plus common-node perturbation, loss-of-function, rescue, and temporal/functional evidence. N axis: extracellular DNA structurally co-localized with CitH3 and neutrophil proteins for NET structures; additional lytic, temporal, and functional evidence for NETotic cell death. | Generalized necrosis; antibody non-specificity; fixation artifacts; non-lytic NET release; mixed-cell neighborhoods | Orthogonal validation, structural controls, cell identity assignment, and predefined evidence threshold | Retain axis-associated terminology; do not claim ferroptosis, PANoptotic mechanism, NET formation, or NETotic cell death as established |
| Clearance/resolution modifiers | Does persistence or resolution modify the relationship between axis activity and injury? | Measure DNase-mediated extracellular-DNA degradation and efferocytic removal separately; quantify persistence longitudinally where feasible | Sampling time, lesion necrosis, impaired access to phagocytes, treatment effects | Treat clearance competence as an unvalidated contextual covariate unless specimen type, normalization, time window, and threshold are prespecified | Do not infer failed clearance or a containment-versus-damage switch |
| Lesion outcome | Does axis-associated activity precede a change in lesion trajectory? | Serial CT/PET-CT; cavity/necrotic-volume change; matrix-injury measures; lesion bacterial burden where feasible; culture conversion; relapse-related outcomes | Regression to the mean; antimicrobial exposure; baseline disease severity; multi-lesion heterogeneity | Longitudinal sampling with prespecified temporal window and lesion-level outcome definition | Cross-sectional correlation only; directionality remains unresolved |
| Causal perturbation | Does selective modulation alter lesion-relevant outcomes? | Selective pharmacological/genetic perturbation with target-engagement verification, bacterial burden, tissue injury, and dissemination readouts | Off-target effects; inadequate lesion exposure; compensatory activation of other injury pathways; host-defense impairment | Appropriate model; vehicle/untreated controls; pharmacokinetic confirmation; rescue or orthogonal perturbation | Marker reduction without outcome change suggests correlate rather than driver |
| Reproducibility and translation | Is the finding robust and relevant beyond one system? | Replication across independent experiments and, where feasible, human tissue plus relevant animal model(s) | Model-specific anatomy, disease stage, drug penetration, assay-platform variation | Explicit source-system reporting and prespecified external-validation plan | Do not generalize lesion mechanism or therapeutic action across systems |

Note. The proposed causal chain is: lesion context → cellular capacity → pathway engagement → mechanism-specific execution → clearance/resolution modifiers → bacterial and tissue outcomes. Arrows represent hypotheses requiring temporal and perturbational testing, not established causal relationships.
